# The Million Veteran Program 1990–1991 Gulf War Era Survey: An Evaluation of Veteran Response, Characteristics, and Representativeness of the Gulf War Era Veteran Population

**DOI:** 10.3390/ijerph21010072

**Published:** 2024-01-08

**Authors:** Kelly M. Harrington, Rachel Quaden, Lea Steele, Drew A. Helmer, Elizabeth R. Hauser, Sarah T. Ahmed, Mihaela Aslan, Krishnan Radhakrishnan, Jacqueline Honerlaw, Xuan-Mai T. Nguyen, Sumitra Muralidhar, John Concato, Kelly Cho, J. Michael Gaziano, Stacey B. Whitbourne, on behalf of the VA Million Veteran Program

**Affiliations:** 1 Million Veteran Program (MVP) Coordinating Center, VA Boston Healthcare System, Boston, MA 02130, USA; rachel.quaden@va.gov (R.Q.); jacqueline.honerlaw@va.gov (J.H.); xuan-mai.nguyen@va.gov (X.-M.T.N.); kelly.cho@va.gov (K.C.); michael.gaziano@va.gov (J.M.G.); stacey.whitbourne@va.gov (S.B.W.); 2 Department of Psychiatry, Boston University Chobanian & Avedisian School of Medicine, Boston, MA 02118, USA; 3 Veterans Health Research Program, Yudofsky Division of Neuropsychiatry, Department of Psychiatry and Behavioral Sciences, Baylor College of Medicine, Houston, TX 77030, USA; lea.steele@bcm.edu; 4 Center for Innovations in Quality, Effectiveness, and Safety (IQuESt), Michael E. DeBakey VA Medical Center, Houston, TX 77030, USA; drew.helmer@va.gov (D.A.H.); sarah.ahmed3@va.gov (S.T.A.); 5 Department of Medicine, Baylor College of Medicine, Houston, TX 77030, USA; 6 VA Cooperative Studies Program Epidemiology Center-Durham, Department of Veterans Affairs, Durham, NC 27705, USA; elizabeth.hauser@va.gov; 7 Department of Biostatistics and Bioinformatics, Duke Molecular Physiology Institute, Duke University, Durham, NC 27705, USA; 8 Cooperative Studies Program Clinical Epidemiology Research Center (CSP-CERC), VA Connecticut Healthcare System, West Haven, CT 06516, USA; mihaela.aslan@va.gov (M.A.);; 9 Department of Internal Medicine, Yale University School of Medicine, New Haven, CT 06511, USA; john.concato@yale.edu; 10 National Mental Health and Substance Use Policy Laboratory, Substance Abuse and Mental Health Services Administration, Rockville, MD 20857, USA; 11 Carle Illinois College of Medicine, University of Illinois, Champaign, IL 61820, USA; 12 Department of Medicine, Harvard Medical School, Boston, MA 02115, USA; 13 Office of Research and Development, Veterans Health Administration, Washington, DC 20420, USA; sumitra.muralidhar@va.gov; 14 Food and Drug Administration, Silver Spring, MD 20993, USA; 15 Division of Aging, Department of Medicine, Brigham and Women’s Hospital, Boston, MA 02115, USA

**Keywords:** Gulf War, Gulf War Illness, cohort studies, environmental exposures, generalizability, health outcomes, Million Veteran Program, Veterans

## Abstract

To address gaps in understanding the pathophysiology of Gulf War Illness (GWI), the VA Million Veteran Program (MVP) developed and implemented a survey to MVP enrollees who served in the U.S. military during the 1990–1991 Persian Gulf War (GW). Eligible Veterans were invited via mail to complete a survey assessing health conditions as well as GW-specific deployment characteristics and exposures. We evaluated the representativeness of this GW-era cohort relative to the broader population by comparing demographic, military, and health characteristics between respondents and non-respondents, as well as with all GW-era Veterans who have used Veterans Health Administration (VHA) services and the full population of U.S. GW-deployed Veterans. A total of 109,976 MVP GW-era Veterans were invited to participate and 45,270 (41%) returned a completed survey. Respondents were 84% male, 72% White, 8% Hispanic, with a mean age of 61.6 years (*SD* = 8.5). Respondents were more likely to be older, White, married, better educated, slightly healthier, and have higher socioeconomic status than non-respondents, but reported similar medical conditions and comparable health status. Although generally similar to all GW-era Veterans using VHA services and the full population of U.S. GW Veterans, respondents included higher proportions of women and military officers, and were slightly older. In conclusion, sample characteristics of the MVP GW-era cohort can be considered generally representative of the broader GW-era Veteran population. The sample represents the largest research cohort of GW-era Veterans established to date and provides a uniquely valuable resource for conducting in-depth studies to evaluate health conditions affecting 1990–1991 GW-era Veterans.

## 1. Introduction

During the 1990–1991 Persian Gulf War (GW), approximately 700,000 U.S. Armed Forces service members were deployed to the Persian Gulf region in support of Operations Desert Shield and Desert Storm. Following deployment, reports described an unexplained multisymptom illness among returning GW Veterans that included chronic headache, widespread pain, persistent fatigue, gastrointestinal distress, skin abnormalities, memory and concentration problems, and mood disturbances [1,2,3,4,5]. This chronic multisymptom illness, commonly referred to as Gulf War Illness (GWI), has affected 25–35% of military personnel deployed in this conflict [6,7,8].

More than three decades after the 1990–1991 Gulf War, research has documented the consistency and persistence of GWI symptoms and associated significant decrements in health and quality of life among GW Veterans [4,9,10,11]. Deployment-related toxic exposures, such as pesticides, pyridostigmine bromide (PB) pills, and chemical agents (e.g., sarin/cyclosarin), have been postulated as invoking a persistent central proinflammatory response among Veterans with GWI [7,12]. Additionally, genetic studies have investigated the role of various genes involved in the development of GWI, and possible genetic risk loci have been identified, including PON1 and BCHE [13,14]; however, replication of these results has proven difficult [15]. Recent systematic reviews of the GWI literature have shown that evidence-based treatments are still lacking and that there are no existing validated biomarkers to identify GWI [16,17]. In summary, significant gaps remain in our understanding of the complex pathophysiology of GWI, particularly with respect to elucidating potential gene-by-environment interactions that may influence susceptibility to GWI. 

The Department of Veterans Affairs (VA) Million Veteran Program (MVP) launched in 2011 with the goal of enrolling at least one million VA users into a genetic and health cohort and has since become one of the largest and most diverse cohorts in the world. Participation in the MVP entails collection of self-reported survey data, provision of a blood specimen for genetic analysis, access to health records, and permission to be recontacted for further data collection or participation in additional research (for details on MVP methodology, see Gaziano et al. [18] and Nguyen et al. [19]).

In 2018, the MVP initiated the first major recontact effort within the program, designed to support the VA Cooperative Studies Program (CSP) #2006 project, “Genomics of Gulf War Illness in Veterans”. A detailed description of the rationale and methods for the CSP#2006 project have been previously published [20,21]. The primary objectives of CSP #2006 are to (1) identify genetic variants associated with GWI and (2) examine interactions between genetic variants and self-reported GW environmental exposures in relation to Veterans’ risk of developing GWI. To achieve these objectives, MVP enrollees who served during the 1990–1991 Gulf War Era were recontacted and invited to complete a comprehensive survey assessing Gulf War-specific deployment characteristics, deployment-related exposures, symptoms of GWI, and comorbid medical conditions. 

The aims of this paper are to (1) describe the methodology used for outreach and data collection from Gulf War Era MVP participants; (2) report survey response rates; (3) compare survey respondents versus non-respondents on demographic, military service, and health-related characteristics; and (4) evaluate the representativeness of the MVP/CSP#2006 Gulf War Veteran cohort relative to the broader U.S. Gulf War Veteran population (overall and among VHA users).

## 2. Materials and Methods

### 2.1. Study Design and Population

#### 2.1.1. MVP 1990–1991 Gulf War Era Survey

At the time of enrollment into the MVP, participants agree to be contacted for additional research purposes. Prior to the full survey implementation, pilot work assessed the feasibility of identifying and contacting Gulf War Era Veterans to complete the survey. Starting in June 2018, MVP enrollees who served during the 1990–1991 Gulf War Era were invited via mail to participate in a survey about their health and their military experiences during 1990–1991. Participants were able to opt out of contact; those who did not opt out were sent the “MVP 1990–1991 Gulf War Era Survey”. The survey questionnaire collected health information that included symptoms associated with GWI, diagnosed medical and psychiatric conditions, healthcare and hospitalization data, lifestyle habits, as well as Gulf War service details such as deployment locations and exposure to agents potentially associated with GWI (see Supplementary File S1 for a copy of the complete survey).

#### 2.1.2. Selection Criteria for Invitational Mailing

Participant selection criteria for the MVP 1990–1991 Gulf War Era Survey were applied in two steps. First, eligible MVP participants (*N* = 589,620) were identified based on the following criteria: (1) an MVP blood specimen was collected; and the participant (2) was still living, (3) had not withdrawn from the MVP, (4) did not opt out of receiving additional research requests, (5) was not included in the pilot work (*n* = 600), and (6) had an accurate and current address on file. Second, data provisioned by the Veterans Affairs/Department of Defense Identity Repository (VADIR) were used to limit the list of eligible MVP participants to only those Veterans with confirmed military service between 1 August 1990 and 31 July 1991 (including Active Duty, Reserves, or National Guard). The MVP and VADIR datafiles were merged by matching social security number (SSN) and date of birth, yielding a total of 109,976 MVP GW-era Veterans who were eligible to be contacted for the survey. This approach identified 24,078 Veterans (21.9%) who had deployed to the 1990–1991 Gulf War and 85,898 Veterans (78.1%) who were in the military during the Gulf War period but had not deployed to the Gulf War theater. 

#### 2.1.3. Overview of Survey Distribution

**Figure 1:**
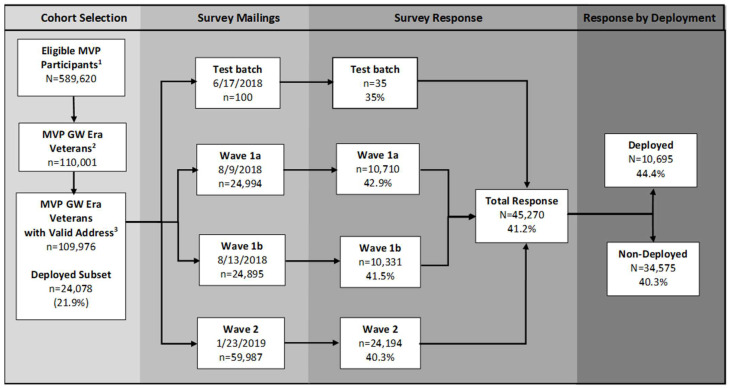
Selection of participants for the MVP 1990–1991 Gulf War Era Survey and response rates. Legend: ^1^ Veterans enrolled in the MVP who gave a blood specimen as of 10 July 2018. ^2^ MVP enrollees with blood specimen who served during 1 August 1990–31 July 1991 (according to VADIR) and who were matched to MVP data using social security number and date of birth. MVP GW Veterans represented 18.7% of all eligible MVP participants enrolled at the time of cohort selection (i.e., 110,001 out of 589,620). ^3^ Mailing vendor had a valid address on file and mailing was not returned due to bad address (*n* = 25 Veterans were excluded due to not having a valid mailing address).

The cohort selection criteria and participant flow diagram for the two major waves of survey mailings are shown in Figure 1. A first wave of 49,989 mailings (Wave 1) took place in the summer of 2018 (i.e., 17 June 2018—13 August 2018). On September 18, 2018, a follow-up mailing (a.k.a. “second invite”) was sent to Veterans who had been contacted for Wave 1 but had not responded (*n* = 36,403). The second wave of 59,987 Gulf War Era Survey mailings (Wave 2) were distributed on January 23, 2019. The follow-up mailing to Wave 2 non-respondents was distributed on March 11, 2019 (*n* = 44,189). Thank-you mailings were sent to all MVP participants who returned surveys. This study has been approved by the VA Central Institutional Review Board (CIRB) and the research oversight/ethics committees at each participating VA facility. 

### 2.2. Measures

#### 2.2.1. Data Sources

In the current paper, the MVP GW Era Survey data were only used to report response rates; that is, no questionnaire item-level responses from the GW Era Survey are reported herein. This section provides a brief description of the four data sources used to evaluate the profile of characteristics of GW Era Survey respondents versus several comparison groups.

1. MVP Baseline Survey: The MVP Baseline Survey collects self-reported information from participants, including demographics, uniformed services experience, activities and habits, health status, medical history, and health care usage. Refer to Nguyen et al. [19] for details regarding MVP Baseline Survey development methods and a compilation of measure source references.
2. VA Corporate Warehouse (CDW): The VA CDW is a structured query language extract from the Veterans Information Systems and Technology Architecture (VISTA) clinical data system. VISTA represents the complete medical record for Veterans Health Administration (VHA) patients and contains records on over 22 million patients, with approximately 12 million patients having had a recorded visit to a VHA facility during the past five years [22].
3. MVP Core Demographics File: This datafile was created by the MVP Data Analytics team and contains participant demographic information at the time of MVP enrollment. Three data sources were compared to determine a Veteran’s best measure for a given demographic variable: MVP Survey data, CDW data, and Observational Medical Outcomes Partnership (OMOP) data; OMOP is a common data model that allows for comparison across disparate observational data sources [23]. If at least two out of three sources matched for a given variable, that value was selected as the final response. In instances of disagreement across sources, the MVP Survey response was given priority whenever available. When MVP Survey responses were unavailable, OMOP was given priority, and if both survey and OMOP responses were missing, CDW data were reported.
4. Veterans Affairs/Department of Defense Identity Repository (VADIR): VADIR provides military service information for Veterans and service members, including branch of service, unit component, military ranks, and deployment dates. This data source is maintained by the Department of Defense (DoD) Manpower Data Center.

#### 2.2.2. Comparison Group Sample Sizes

Demographic, military, and health-related characteristics were compared between GW Era Survey respondents and three comparison groups, as defined below: (1) GW Era Survey non-respondents, (2) GW-era VHA users, and (3) the entire population of U.S. deployed 1990–1991 GW Veterans. The first comparison group was selected to allow for an examination of differences between survey respondents and non-respondents, while comparison groups 2 and 3 were selected to evaluate whether the MVP/CSP#2006 Gulf War Veteran cohort is representative of the broader GW-era VHA user population and the full Gulf War Veteran population, respectively.

1. Comparison group 1 (CG-1): All 109,976 Veterans who were mailed a GW Era Survey were used to evaluate response rates in relation to military and demographic characteristics (*n* = 45,270 GW Era Survey respondents and *n* = 64,706 non-respondents). When examining health-related characteristics, the cohort was limited to those who had completed the MVP Baseline Survey (*n* = 40,040 GW Era Survey respondents and *n* = 29,827 non-respondents).
2. Comparison group 2 (CG-2): The GW-era VHA user sample included all living Veterans at the time of mailing (15 November 2018) who had at least one VHA visit and had served during the 1990–1991 GW Era according to military service information in VADIR (*n* = 1,751,873). Although GW Era Survey respondents (*n* = 45,270) represent a subset of CG-2, they were excluded from CG-2 to allow for analysis of independent samples.
3. Comparison group 3 (CG-3): GW Era Survey respondents who were deployed to the Gulf theater between 1 August 1990 and 31 July 1991, (*n* = 10,695) were compared to the entire population of U.S. deployed 1990–1991 GW Veterans (*n* = 696,470) [24].

#### 2.2.3. Demographic, Military Service, and Health-Related Characteristics

1. Demographic variables: When comparing GW Era Survey respondents to non-respondents (CG-1), and to all deployed GW Veterans (CG-3), gender, age, race, and ethnicity were determined primarily based on the MVP Core Demographics file. For GW-era VHA users (CG-2), demographic characteristics were primarily based on CDW data. For individuals who were missing data in both MVP Core Demographics and CDW files, values were supplemented with VADIR information whenever available. Age in years was calculated at the date of the GW Survey mailing (i.e., date of first mailing or 15 November 2018, which represents the midpoint between batches of first mailed survey invites, for those who were not invited to participate in the GW Survey). Ages outside of 44 through 96 years were set to missing as they were unlikely to be correct for Veterans serving during the 1990–1991 GW Era. Marital status and whether Veterans had been designated by the VA to have military service-connected disabilities were reported from CDW. For the subset of CG-1 with a completed MVP Baseline Survey, education and income were also reported.
2. Military service variables: Service branch, deployment during the GW Era, unit component (i.e., Active duty, National Guard, or Reserves), and military rank (i.e., enlisted, officer, or warrant officer) in or closest to August 1990 were reported from VADIR. The data cleaning procedure used for defining military rank was previously described in detail by Duong and colleagues [21]. Veterans’ history of serving in a combat zone was reported from CDW.
3. Health-related variables: Health-related characteristics were reported for GW Era Survey respondents and non-respondents with a completed MVP Baseline Survey (*n* = 69,867) including current health status (“good to excellent”, “fair to poor”), smoking status (“never”, “former”, “current”), current physical fitness status (“very good to fairly good”, “satisfactory”, “fairly poor to very poor”), current exercise frequency (“≤1–3 times/month”, “once/week”, “2–4 times/week”, “≥5 times/week”), pain intensity in the past week (scale from 0 to 10), VA health care use in the past year (“none”, “less than half”, “more than half”, “all care”), and number of VA inpatient hospital stays in the past year. The Veterans RAND 12 Item Health Survey (VR-12) [25,26] is a standardized index of general health and quality of life developed for use in Veteran populations, and it was included in the MVP Baseline Survey. We reported the VR-12 physical component score (PCS) and mental component score (MCS), calculated using a SAS macro provided by the developer, Dr. Lewis Kazis [27]. For both the VR-12 PCS and MCS, the population mean is 50 (*SD* = 10) and higher scores correspond to better health-related quality of life. To assess alcohol use, we used the Alcohol Use Disorders Identification Test (AUDIT-C) which was included in the MVP Baseline Survey [28]. The AUDIT-C was scored and interpreted following standard procedures, whereby an individual is considered to screen positively for problematic alcohol use with a score of 4 or more for men and 3 or more for women. The MVP Baseline Survey also asks participants if they were “ever diagnosed” with a list of 75 health conditions. Participants were considered to have a health condition if they answered ‘yes’ to the diagnosis question, provided a year of diagnosis, or checked they are currently taking medication for the given condition. We reported the mean of the total number of heath conditions that each participant answered affirmatively. The Charlson Comorbidity Index (CCI) was developed to assess mortality risk by weighting 17 comorbidities [29]. A CCI score of zero indicates that a patient does not have any of the 17 comorbid conditions assessed, and higher CCI scores correspond to an increasing predicted mortality rate. For the current study, the CCI was calculated for each individual using International Classification of Diseases (ICD-9/10) code diagnosis data for the 17 conditions obtained from CDW between 16 November 2008, and 15 November 2018. Body mass index (BMI) was computed generally following the methods (including height and weight cleaning procedures) described by Nguyen et al. [30]. Weight measurements were obtained from CDW between 16 November 2016, and 15 November 2020 and height measurements between 16 November 2013 and 15 November 2023 (i.e., representing a +/− 2-year window and +/− 5-year window, respectively, from the midpoint of MVP GW Survey data collection: 15 November 2018).

### 2.3. Data Analysis

First, we calculated survey response rates according to survey wave (i.e., 2018 vs. 2019) and deployment status (i.e., deployed vs. not deployed). Veterans who returned partially complete surveys were counted as survey respondents whereas those who returned totally blank surveys (*n* = 337) were counted as non-respondents. Second, we generated frequency tables to compare demographic, military, and health characteristics between GW Survey respondents and the three comparison groups outlined above. We also calculated the standardized mean difference (SMD/Cohen’s *d*) to determine whether there were significant differences between respondents and non-respondents on each of the characteristics examined [31,32,33]. Given the large sample size of this study, we chose to report SMD as a measure of the magnitude of group differences because large samples render *p*-values considerably less meaningful [34,35]. According to Andrade [35], SMDs of 0.2, 0.5, and 0.8 are considered small, medium, and large, respectively. Finally, we conducted stratified analyses to determine whether the marked differences in response rates observed between GW officers vs. enlisted personnel may have driven apparent differences in other response characteristics (i.e., whether the observed patterns may be due to confounding by military rank). All analyses were conducted in SAS 9.4 (SAS Institute Inc., Cary, NC, USA).

## 3. Results

### 3.1. Survey Response Rate

Among the 109,976 MVP enrollees who served during the GW Era and were invited to participate in the 1990–1991 Gulf War Era Survey, 45,270 had completed and returned a survey by 28 October 2021. This represents an overall survey response rate of 41.2%, with Veterans deployed to the GW region showing a modestly higher response rate than their non-deployed counterparts (44.4% vs. 40.3%, respectively; see Figure 1). The survey response rate was consistent across the two major waves of invitational mailings (range: 40.3–42.9%).

### 3.2. Characteristics of Gulf War Era Survey Respondents Versus Non-Respondents

Compared with non-respondents, GW Era Survey respondents were older, on average, and included higher proportions of Veterans who were White, were married or cohabitating with a partner, were officers, had served in the Air Force, and were deployed to the Persian Gulf region (see Table 1). Survey respondents were also more likely to report higher annual income and level of education than non-respondents. In contrast, non-respondents were younger, more racially and ethnically diverse, and included a higher proportion of enlisted and Army personnel. Respondents and non-respondents had a similar gender distribution and component distribution, and the two groups did not differ with respect to mean BMI level. The largest differences (i.e., SMDs) were observed for age, race, and military rank.

Among the subset of MVP GW-era Veterans who had previously completed the MVP Baseline Survey (*n* = 69,867), health-related characteristics were compared between GW Era Survey respondents (*n* = 40,040) vs. non-respondents (*n* = 29,827) (see Table 2). The proportion of GW Survey respondents with a Baseline Survey was nearly double that of non-respondents with a Baseline Survey (88.4% vs. 46.1%). Respondents appeared to be somewhat healthier, overall, than non-respondents. Specifically, higher proportions of respondents reported “good to excellent” health (i.e., 66.6% vs. 57.0%) and “very good to fairly good” current physical fitness status (i.e., 37.9% vs. 30.0%). In addition, respondents reported higher weekly exercise frequency and lower pain levels in the past week than non-respondents. Non-respondents reported receiving more of their healthcare from the VA than respondents (i.e., 65.2% vs. 58.1% received at least half of their care from VA). More non-respondents also reported VA inpatient stays in the past year than respondents (i.e., 16.9% vs. 11.5% had one or more VA inpatient stays). Non-respondents had slightly lower scores on both the physical component summary and mental component summary scores on the VR-12 relative to respondents. A greater proportion of non-respondents reported current smoking than respondents (23.1% vs. 15.7%). Conversely, a slightly higher proportion of respondents screened positive for hazardous drinking on the AUDIT-C compared to non-respondents. The two groups did not differ in the mean number of self-reported health conditions or the Charlson Comorbidity Index. Notably, the differences (SMDs) were small for all health characteristics compared between the two groups. 

### 3.3. Stratified Analysis of Survey Response Rates by Deployment Status and Rank

Given the sizable observed differences in response rates between officers and enlisted personnel, we conducted a stratified analysis of response rates by deployment status and rank. Complete results can be accessed in Supplementary File S2. In summary, officers had consistently higher response rates than enlisted personnel across demographic, military, and health strata and in both deployed and non-deployed Veterans. There was evidence to support that associations of demographic and military characteristics with survey response frequencies typically occurred in similar patterns, independent of military rank. That is, a pattern of higher response in relation to GW deployment, older age, and better health appears in both officers and enlisted groups. There were a few variables, however, in which response differences did appear to be correlated with military rank (e.g., race/ethnicity, military branch). For example, differences in response frequencies between racial subgroups were more pronounced in officers than in enlisted personnel, for both deployed and non-deployed Veterans (i.e., deployed White officers had ~32% higher response than deployed Black officers, whereas deployed White enlisted personnel had ~14% higher response than Black enlisted personnel).

### 3.4. Comparison of Gulf War Era Veteran Survey Respondents to All Gulf War Era VHA Users

Compared with the overall population of living GW-era VHA users at the time of the mailing, GW Era Survey respondents included a higher proportion of women (16% vs. 12%), were four years older on average (61.6 vs. 57.5 years), and were less racially diverse (e.g., 20% vs. 24% identified as Black/African American). Survey respondents also included higher proportions of Veterans who were married (64% vs. 55%), were military officers (21% vs. 13%), had VA service-connected disabilities (85% vs. 74%), and had served in a combat zone (18% vs. 13%) (see Table 3). Among GW Era Survey respondents, there was slightly more representation from the Army and Reserves, and the mean score on the Charlson Comorbidity Index was slightly higher relative to the broader GW-era population receiving VHA healthcare.

### 3.5. Comparison of Deployed Gulf War Veteran Survey Respondents to All Deployed Gulf War Veterans

Finally, the deployed GW Era Survey respondents (*n* = 10,695) were also compared to the entire population of U.S. Veterans who served in the 1990–1991 Gulf War (*n* = 696,470) as documented in a 2002 federal report (see Table 4) [24]. SMD scores were not calculated for this comparison because the deployed GW Era Survey respondents (*n* = 10,695) are a subset of the entire population of GW Veterans *(n* = 696,470). Survey respondents were very similar to the overall population of GW Veterans, with limited differences. The deployed GW Survey respondents were more likely to be female, and were about three years older, on average, at their time of deployment (i.e., age in 1991) compared with the full population of GW Veterans. Additionally, deployed GW Survey respondents were more likely to have served in the Army, to be in the Reserves or National Guard, and to be officers compared with the full population of GW Veterans. Notably, the racial composition of the two groups was nearly identical.

## 4. Discussion

The MVP 1990–1991 Gulf War Era Survey represents a successful first large-scale recontact effort of MVP participants. The resulting sample represents the largest research cohort of 1990–1991 Gulf War and non-deployed Gulf War-Era Veterans collected to date. In addition to standard assessment of survey response rates and characteristics, available data allowed detailed comparisons of the final study sample to the entire patient population of VHA users who served during the 1990–1991 Gulf War Era, as well as the full population of U.S. Veterans who served in the 1990–1991 Gulf War. The overall comparability of the MVP Gulf War Era study cohort to the larger populations of U.S. Gulf War Veterans and Veterans of the era, together with the multifaceted data resources assembled for the project, provide an important foundation for detailed studies of long-term health consequences associated with the 1990–1991 Gulf War. 

The overall MVP GW Survey response rate of 41% is comparable to response rates observed for other large government-sponsored Veteran population surveys. It is similar to that of the recent MVP COVID-19 Survey distributed via mail in 2020 (40% response rate) [36] and higher than the Mental Health Questionnaire participation rate of 31% for the original UK Biobank participants [37]. In addition, it is not far from the 53% response rate observed for the mailed portion of the population-based survey of 30,000 Gulf War Veterans conducted in 1995 [6] and higher than the response rate of 34% for the first follow-up survey of the 1995 cohort of Gulf War Veterans [38].

Survey response rates were modestly higher among deployed vs. non-deployed Veterans (44% vs. 40%). Response was most consistently higher among older Veterans, those who identified as White, and Veterans who had served as officers in the military. Additional stratified analyses were undertaken to determine whether the elevated response rate among officers may be driving differences in other response characteristics. We concluded that rank-related response differences did not generally confound the overall pattern of demographic, military, and health associations with response rates, although response patterns observed across racial/ethnic subgroups and military branches differed for officers vs. enlisted personnel.

Survey respondents also reported better health than non-respondents on a variety of indicators, although the magnitude of health differences was uniformly small. This included more favorable self-reported current health status, physical fitness, exercise frequency, and pain levels. However, on average, respondents and non-respondents showed a similar number of self-reported specific health conditions and a comparable degree of medical comorbidity based on VA electronic health record data (as indicated by the Charlson Comorbidity Index). And, although survey respondents were less likely to be current smokers than non-respondents, they were slightly more likely to screen positive for problematic drinking. Survey respondents also had slightly higher scores on both the physical and mental component scales of the VR-12 relative to non-respondents, indicating higher health-related quality of life. Regarding patterns of healthcare utilization, survey respondents were less likely to rely predominantly on VA health care than non-respondents. This finding is in line with the higher on-average levels of education and income reported by respondents, suggesting they may have slightly greater access to private insurance and non-VA healthcare services than non-respondents. 

In this paper, we also compared GW Era Survey respondents to the overall population of 1990–1991 GW-era Veterans using the VHA system at the time of data collection. We found that the demographic characteristics of the survey respondents were generally consistent with those of the VHA-enrolled GW-era population, with minor differences. Notably, GW Era Survey respondents included a higher proportion of Veterans who were women, were married, and were military officers than the GW-era VHA user population; respondents were also slightly older and less racially diverse than the GW-era VHA user population. A similar pattern was observed in the UK Biobank Study, in that the participation rate was higher among women, older age groups, and persons living in less socioeconomically marginalized areas compared with the general population [39]. GW Era Survey respondents were also more likely to have served in a combat zone, to have a VA service-connected disability rating, and had a slightly greater level of medical comorbidity (i.e., higher Charlson Comorbidity Index) than the overall GW-era population using VHA services. Taken together, the higher frequencies of service in a military combat zone, VA service-connected disability, and medical comorbidity observed among GW Era Survey respondents suggests they may carry a slightly higher disease burden than the larger population of all GW-era VHA users. Alternatively, the higher degree of medical comorbidity seen in respondents (per the CCI) may be an artifact of their higher healthcare utilization, providing more opportunity for documenting ICD diagnostic codes. These findings are generally consistent with previous studies that have identified motivational factors that influence Veterans’ participation in health research, including altruism, giving back to the medical community for the care they have received, and a desire to help scientists learn how to treat other Veterans with the same disease, which seem to be particularly strong among Veterans with armed combat experience [40,41,42].

Perhaps most relevant to study objectives, we were able to compare key characteristics of GW-deployed MVP Survey respondents to the full population of 696,470 deployed U.S. GW Veterans. The MVP GW sample generally was very similar to the full population of U.S. 1990–1991 GW Veterans. The racial composition of the two groups was nearly identical, while the MVP GW Veteran study cohort was, on average, three years older and included slightly higher proportions of women, officers, Reservists, and Army Veterans compared with the full population of U.S. 1990–1991 GW Veterans. Overall similarities demonstrate that the MVP cohort of GW-era Veterans appears to be generally representative of the broader GW-era Veteran population, including those receiving care in the VA healthcare system. 

Finally, the health profile of GW Era Survey respondents was slightly more favorable than non-respondents with respect to a range of self-reported health indicators (e.g., higher perceived physical health and fitness level, greater exercise frequency, lower pain levels) as well as socioeconomic status indicators (e.g., higher educational attainment, higher income level). This finding is suggestive of a possible “healthy volunteer” response bias in this GW-era cohort, which has been well-documented in other volunteer-based cohort studies [39,43,44]. Although such response bias appears to have occurred to some extent in the current GW-era cohort, it differs from prior evidence that MVP participants, overall, are not healthier than non-VHA Veterans or the general U.S. population. For example, previous studies have shown that compared with the general population, MVP participants have a higher prevalence of overweight and obesity and are more likely to be seeking health care [30]. We similarly found that the mean BMI for both MVP GW Survey respondents and non-respondents was ~31 kg/m^2^, falling in the Class I obese category according to clinical guidelines [45], which is higher than the mean BMI level reported in the general U.S. population (c.f., National Health and Nutrition Examination Survey [NHANES]; [46]).

### Strengths and Limitations

A major strength of the current study is our use of multiple data sources that were external to the MVP GW Era Survey data to evaluate the characteristics of the study cohort in relation to several key comparison groups (i.e., GW Survey non-respondents, GW-era VHA users, and the full U.S. population of 1990–1991 GW Veterans). This detailed investigation is critically informative regarding the generalizability of results generated using this cohort. The overall similarity of the MVP GW-era sample with GW-era VHA users and with all U.S. Gulf War Veterans provides confidence that research findings from this cohort indeed are likely to be representative of Gulf War Veterans.

Other important strengths of this study include the assembly and detailed characterization of the largest sample of deployed 1990–1991 GW Veterans and non-deployed GW-era Veteran cohorts. It provides good representation of subpopulations of GW-era Veterans (e.g., gender, race, ethnicity, age, education, military branch), which affords greater power to evaluate differences across subgroups than in previous GW studies. Data for the study were collected in 2018–2019, providing health and related information on Veterans of the 1990–1991 GW Era 28 years after the war. This aspect is particularly important, as studies have indicated that the health of GW Veterans has continued to decline in the decades after the war as Veterans have aged [9,47].

We acknowledge several limitations inherent in volunteer-based survey studies, including participation bias, recall bias (particularly when reporting on military experiences from nearly 30 years ago), and reporting bias (e.g., over- or under-reporting of symptoms) which may reduce the accuracy and generalizability of certain data and results in studies conducted using this cohort. The expected impact of some of these biases are more predictable than others. For example, if the accuracy of Veteran-reported exposures is “nondifferential” in relation to their health status, identified health-exposure associations would be biased towards the null, potentially obscuring accurate identification of true associations. Conversely, if sicker Veterans routinely “over report” a given exposure more commonly than healthy Veterans, the estimated health–exposure association would be inflated. Such limitations have long been associated with GW Veteran population research and have potentially increased over time in some respects. In addition, government and research sources have characterized the U.S. population of non-deployed GW-era Veterans in different ways for different purposes since 1991. We did not identify a report that described the overall U.S. population of non-deployed GW-era Veterans that was suitable for current purposes. Therefore, we could not directly compare our non-deployed GW-era Survey respondents to the appropriate population.

## 5. Conclusions

The VA MVP/CSP #2006 study population represents the largest cohort of 1990–1991 GW-era Veterans available for research, laying the foundation for future analyses of the complex relationships among GWI phenotypes, genes, and GW deployment exposures. This cohort offers a valuable resource for future epidemiologic and genomic studies investigating GWI and other health outcomes in Veterans, important work that is already underway (e.g., Radhakrishnan et al. [20]; Duong et al. [21]; Koller et al. [48]). We demonstrated here that the demographic, military, and health characteristics of this GW-era Veteran sample are largely consistent with the general population of deployed GW Veterans and GW-era Veterans using VHA healthcare services and can be considered generally representative of the broader GW-era Veteran population. Future investigations based on this sample are expected to provide reasonably unbiased estimates of genetic and exposure–disease associations that are generalizable to the GW Veteran population.

## Figures and Tables

**Table 1:** Demographic and military characteristics of Gulf War Era Veteran Survey respondents vs. non-respondents.

Characteristics	All (*N* = 109,976)	Respondents (*n* = 45,270)	Non-Respondents (*n* = 64,706)	SMD ^8^
Gender ^1^ (%)				0.01 *
Male	83.5	83.7	83.5	
Female	16.5	16.3	16.5	
Age ^1^				0.48 **
Mean (*SD*)	59.3 ± 8.4	61.6 ± 8.5	57.7 ± 7.9	
Median (*Q1*, *Q3*)	58 (52, 65)	61 (55, 68)	56 (51, 63)	
Minimum–Maximum	44–94	45–94	44–93	
Age Group ^1^ (%)				0.46 **
40–49	12.0	7.5	15.2	
50–59	43.0	35.4	48.4	
60–69	30.9	37.1	26.6	
70+	14.0	20.0	9.7	
Race ^1^ (%)				0.36 **
White	63.7	72.1	57.9	
Black/African American	27.6	18.6	33.8	
American Indian/Alaskan Native	0.9	0.8	0.9	
Asian ^5^	1.5	1.4	1.6	
Other	2.5	2.7	2.3	
Multiple Responses	3.5	4.1	3	
Unknown	0.4	0.2	0.6	
Ethnicity ^1^ (%)				0.04 *
Hispanic ^6^	8.9	8.3	9.3	
Non-Hispanic	90.7	91.5	90.2	
Unknown	0.4	0.1	0.5	
Education ^2^				0.26 **
High school or less	11.7	10.2	13.8	
Some college	28.8	26.3	32.2	
Associates	15.9	15.4	16.6	
Bachelors	21.3	22.3	20.1	
Masters/Professional/Doctorate	20.9	24.8	15.7	
Missing	1.3	1.1	1.7	
Income (annual) ^2^				0.29 **
<$30,000	18.6	14.8	23.6	
$30,000–$59,999	28.1	27.1	29.4	
$60,000–$99,999	24.5	26.1	22.2	
$100,000+	18.9	21.8	15.0	
Missing	10.0	10.3	9.6	
Marital Status ^3^ (%)				0.11 *
Married/cohabitating with partner	57.3	64.3	51.4	
Missing	2.6	3.0	2.3	
Service Branch ^4^ (%)				0.13 *
Army	53.2	52.0	54.0	
Navy	21.1	20.2	21.7	
Air force	17.5	20.0	15.7	
Marine Corps	7.0	6.3	7.6	
Coast Guard	1.1	1.3	1.0	
Other ^7^	0.2	0.2	0.1	
Deployment to Persian Gulf Region in 1990–1991 ^4^ (%)				0.07 *
Yes	21.9	23.6	20.7	
No	78.1	76.4	79.3	
Rank ^4^ (%)				0.36 **
Enlisted	82.7	75.1	88.1	
Officer	13.6	20.5	8.7	
Warrant Officer	1.9	2.7	1.4	
N/A or Missing	1.8	1.6	1.9	
Component ^4^ (%)				0.04 *
Active Duty	61.5	61.3	61.6	
National Guard	12.2	12.9	11.8	
Reserves	26.3	25.8	26.7	
BMI ^3^				−0.07 *
Mean (*SD*)	31.4 (5.9)	31.2 (5.7)	31.6 (6.1)	
Missing (%)	9.1	10.4	8.2	

^1^ Data source: Core demographics file supplemented with VADIR. ^2^ Data source: MVP Baseline Survey. Percentages out of those that completed a baseline survey (all *n* = 69,867, respondents *n* = 40,040, non-respondents *n* = 29,827). ^3^ Data source: CDW. ^4^ Data source: VADIR. ^5^ Asian includes Chinese, Japanese, Asian Indian, Other Asian, Filipino, and Pacific Islander. ^6^ Hispanic includes Mexican, Puerto Rican, Cuban, other Spanish/Hispanic/or Latino/or multiple responses. ^7^ Other includes Coast Guard, National Oceanic and Atmospheric Administration (NOAA), Public Health Service (PHS), and multiple branches. ^8^ Standardized mean differences (SMD) exclude missing values. * = small effect size, ** = medium effect size.

**Table 2:** Health-related characteristics of Gulf War Era Veteran Survey respondents vs. non-respondents as reported in the MVP Baseline Survey (*n* = 69,967).

Characteristics	Respondents (*n* = 40,040)	Non-Respondents (*n* = 29,827)	SMD ^1^
Current health status (%)			0.20 *
Good to excellent	66.6	57.0	
Fair to poor	33.1	42.4	
Missing	0.3	0.6	
Current physical fitness status (%)			0.19 *
Very good to fairly good	37.9	30.0	
Satisfactory	36.2	36.8	
Fairly poor to very poor	25.3	32.3	
Missing	0.6	0.9	
Current exercise frequency (%)			0.12 *
≤1–3 times/month	38.8	44.3	
Once/week	14.2	14.0	
2–4 times/week	32.7	28.7	
≥5 times/week	13.6	11.7	
Missing	0.8	1.3	
Pain intensity, past week (%)			−0.19 *
No pain (0)	9.8	8.2	
Mild pain (1–3)	36.1	28.7	
Moderate pain (4–6)	29.3	30.7	
Severe pain (7–10)	24.5	31.9	
Missing	0.3	0.5	
VA health care use, past year (%)			0.17 *
None	11.8	8.6	
Less than half of care	29.1	24.6	
More than half of care	24.6	25.3	
All care	33.5	39.9	
Missing	1.1	1.7	
VA inpatient hospital stays, past year (*n*)			0.18 *
None	78.8	72.7	
1–3	9.7	13.6	
≥4	1.8	3.3	
Missing	9.8	10.4	
VR-12 score (mean, *SD*)			
PCS	36.7 ± 12.1	34.7 ± 12.3	0.16 *
MCS	46.3 ± 13.3	42.5 ± 14.1	0.27 *
Smoker			0.19 *
Never	42.2	38.7	
Former	40.8	36.6	
Current	15.7	23.1	
Missing	1.3	1.6	
Drinker (AUDIT-C) ^2^			0.05 *
Yes	22.0	19.9	
No	75.9	76.7	
Missing	2.2	3.4	
Charlson Comorbidity Index ^3^			0.04 *
Mean (*SD*)	3 ± 2.1	3 ± 2.5	
Median (*Q1*, *Q3*)	3 (2, 4)	2 (1, 4)	
Missing	0.1	0.2	
Number of Health Conditions ^4^			0.02 *
Mean (*SD*)	7.1 ± 3.8	7.2 ± 4.1	
Median (*Q1*, *Q3*)	7 (4, 9)	7 (4, 10)	
Missing	12.4	12.9	

Note: Table represents the subset of GW Veterans we mailed to who responded to the MVP Baseline Survey. Unless noted, all responses are from the Baseline Survey. ^1^ Standardized mean differences (SMD) exclude missing values. * = small effect size. ^2^ Drinker—based on the AUDIT-C: defined as a positive screen for a score of 4 or more for males and a score of 3 or more for females. ^3^ Charlson Comorbidity Index calculated from data in CDW. ^4^ Number of positive responses to 75 conditions listed on the MVP Baseline Survey.

**Table 3:** Characteristics of Gulf War Era Veteran Survey Respondents vs. All Gulf War Era VHA Users.

Characteristics	GW Survey Respondents(*n* = 45,270)	GW-Era VHA Users ^1^ (*n* = 1,751,873)	SMD ^5^
Gender ^2^ (%)			0.12 *
Male	83.7	87.9	
Female	16.3	12.1	
Age ^2^			0.50 **
Mean (*SD*)	61.6 ± 8.4	57.5 ± 8.1	
Median	61	56	
Min–Max	44–93	44–96	
*Q1*,* Q3*	55, 68	51, 63	
Missing	<11	546	
Race ^2^ (%)			0.13 *
White	76.4	69.9	
Black/African American	19.5	24.1	
Asian	1.3	2.1	
American Indian/Alaskan Native	0.7	0.8	
Other	0.9	1.0	
Multiple Responses	0.8	0.7	
Missing	0.4	1.4	
Ethnicity ^2^ (%)			−0.02 *
Hispanic or Latino	6.1	6.3	
Not Hispanic or Latino	93.1	89.1	
Unknown	0.8	4.6	
Marital Status ^3^ (%)			0.08 *
Married/cohabitating with partner	64.3	55.2	
Missing	3.0	11.3	
Service Branch ^4^ (%)			0.10 *
Army	52.0	47.4	
Navy	20.2	21.1	
Air force	20.0	21.0	
Marine Corps	6.3	7.8	
Coast Guard	1.3	1.3	
Other	0.2	1.4	
Rank ^4^ (%)			0.23 **
Enlisted	75.1	85.4	
Officer	20.5	12.5	
Warrant Officer	2.7	2.0	
N/A or Missing	1.6	0.1	
VA Service-Connected Disability ^3^ (%)			0.27 **
Yes	84.9	73.9	
No	15.1	26.1	
Mean (*SD*) Disability Rating %	63.6 ± 32.2	56.8 ± 34.0	0.21 **
Ever Served in Combat Zone ^3^ (%)			0.09 *
Yes	17.5	12.5	
No	66.4	59.9	
Unknown	16.2	27.5	
Component ^4^ (%)			0.10 *
Active Duty	61.3	66.3	
National Guard	12.9	11.2	
Reserves	25.8	22.5	
Charlson Comorbidity Index ^3^			0.59 ***
Mean (*SD*)	3 (2.1)	1.9 (1.7)	
Median (*Q1*, *Q3*)	3 (2, 4)	1 (1, 3)	

^1^ Veterans who were alive as of 15 November 2018, who had at least one inpatient or outpatient VHA visit, and had served during the 1990–1991 GW Era according to VADIR. This comparison group excludes the GW Survey respondents (*n* = 45,270). ^2^ Data source: CDW supplemented with VADIR. ^3^ Data source: CDW. ^4^ Data source: VADIR. ^5^ Standardized mean differences (SMD) exclude missing values. * = small effect size, ** = medium effect size, *** = large effect size.

**Table 4:** Demographic and military characteristics of deployed MVP GW Survey sample and the full U.S. population of 1990–1991 Gulf War Veterans.

Demographic Characteristics	Deployed MVP Gulf War Survey Respondents ^1^ (*n* = 10,695)	All Deployed Gulf War Veterans ^2^ *n* = 696,470
Gender (%)		
Male	88.9	92.5
Female	11.1	7.2
Unknown	0.0	0.3
Age Group in 1991 (%)		
Mean age in years (*SD*)	31.4 ± 7.8	28.0
<25	25.5	40.9
25–34	42.0	40.3
35–44	27.7	15.6
45–54	4.5	2.6
55–64	0.2	0.2
≥65	0.0	0.0
Unknown	0.0	0.4
Race (%)		
White	66.5	67.7
Black/African American	23.5	22.6
American Indian/Alaskan Native	0.9	0.6
Asian ^3^	1.6	2.3
Other	3.1	1.4
Multiple Responses	4.2	--
Unknown	0.2	0.4
Ethnicity (%)		
Hispanic ^4^	9.8	5.1
Non-Hispanic	90.1	--
Unknown	0.1	--
Service Branch (%)		
Army	61.0	50.4
Air Force	10.4	11.9
Marine Corps	11.3	14.9
Navy	17.0	22.7
Coast Guard	0.2	0.1
Rank in August 1990–1991 (%)		
Enlisted	82.3	89.3
Officer	13.7	9.5
Warrant Officer	3.0	1.2
Component (%)		
Active Duty	76.9	83.9
Reserve & National Guard	23.1	16.1

^1^ Data sources for deployed GW Survey respondents: gender, age (calculated as of 1 January 1991, to be consistent with the VA/DoD report), race, ethnicity from MVP Core demographic file supplemented with VADIR data; service branch, rank, and component from VADIR. ^2^ Responses from VA/DoD report. Includes all military personnel deployed to the Gulf theater between 1 August 1990 and 31 July 1991. Note: VA/DoD report combined race and ethnicity in a single variable, adding to 100%. Ethnicity is shown separately in this table to align with how data were collected for deployed MVP GW Veterans, but the two sources are not entirely comparable. ^3^ Asian includes Chinese, Japanese, Asian Indian, Other Asian, Filipino, and Pacific Islander (definition applies only to Deployed Gulf War Survey Respondents). ^4^ Hispanic includes Mexican, Puerto Rican, Cuban, other Spanish/Hispanic/or Latino/or multiple responses (definition applies only to MVP Deployed Gulf War Survey Respondents).

## Data Availability

Due to the nature of this research, participants of this study did not agree for their data to be shared publicly, so supporting data are not available.

## Supplementary Materials

The following supporting information can be downloaded at: https://www.mdpi.com/article/10.3390/ijerph21010072/s1, Supplementary File S1: MVP 1990–1991 Gulf War Era Survey; Supplementary File S2: Gulf War Era Veteran Response Rates Stratified by Deployment Status and Rank.

## Appendix A. VA Million Veteran Program: Core Acknowledgement for Publications February 2023

**MVP Program Office**

- Sumitra Muralidhar, Ph.D., Program Director
- US Department of Veterans Affairs, 810 Vermont Avenue NW, Washington, DC 20420
- Jennifer Moser, Ph.D., Associate Director, Scientific Programs
- US Department of Veterans Affairs, 810 Vermont Avenue NW, Washington, DC 20420
- Jennifer E. Deen, B.S., Associate Director, Cohort & Public Relations
- US Department of Veterans Affairs, 810 Vermont Avenue NW, Washington, DC 20420

**MVP Executive Committee**

- Co-Chair: Philip S. Tsao, Ph.D.
- VA Palo Alto Health Care System, 3801 Miranda Avenue, Palo Alto, CA 94304
- Co-Chair: Sumitra Muralidhar, Ph.D.
- US Department of Veterans Affairs, 810 Vermont Avenue NW, Washington, DC 20420
- J. Michael Gaziano, M.D., M.P.H.
- VA Boston Healthcare System, 150 S. Huntington Avenue, Boston, MA 02130
- Elizabeth Hauser, Ph.D.
- Durham VA Medical Center, 508 Fulton Street, Durham, NC 27705
- Amy Kilbourne, Ph.D., M.P.H.
- VA HSR&D, 2215 Fuller Road, Ann Arbor, MI 48105
- Shiuh-Wen Luoh, M.D., Ph.D.
- VA Portland Health Care System, 3710 SW US Veterans Hospital Rd, Portland, OR 97239
- Michael Matheny, M.D., M.S., M.P.H.
- VA Tennessee Valley Healthcare System, 1310 24th Ave. South, Nashville, TN 37212
- Dave Oslin, M.D.
- Philadelphia VA Medical Center, 3900 Woodland Avenue, Philadelphia, PA 19104

**MVP Co-Principal Investigators**

- J. Michael Gaziano, M.D., M.P.H.
- VA Boston Healthcare System, 150 S. Huntington Avenue, Boston, MA 02130
- Philip S. Tsao, Ph.D.
- VA Palo Alto Health Care System, 3801 Miranda Avenue, Palo Alto, CA 94304

**MVP Core Operations**

- Lori Churby, B.S., Director, MVP Regulatory Affairs
- VA Palo Alto Health Care System, 3801 Miranda Avenue, Palo Alto, CA 94304
- Stacey B. Whitbourne, Ph.D., Director, MVP Cohort Management
- VA Boston Healthcare System, 150 S. Huntington Avenue, Boston, MA 02130
- Jessica V. Brewer, M.P.H., Director, MVP Recruitment & Enrollment
- VA Boston Healthcare System, 150 S. Huntington Avenue, Boston, MA 02130
- Shahpoor (Alex) Shayan, M.S., Director, MVP Recruitment and Enrollment Informatics VA Boston Healthcare System, 150 S. Huntington Avenue, Boston, MA 02130
- Luis E. Selva, Ph.D., Executive Director, MVP Biorepositories
  VA Boston Healthcare System, 150 S. Huntington Avenue, Boston, MA 02130
- Saiju Pyarajan Ph.D., Director, Data and Computational Sciences
  VA Boston Healthcare System, 150 S. Huntington Avenue, Boston, MA 02130
- Kelly Cho, M.P.H, Ph.D., Director, MVP Phenomics Data Core
  VA Boston Healthcare System, 150 S. Huntington Avenue, Boston, MA 02130
- Scott L. DuVall, Ph.D., Director, VA Informatics and Computing Infrastructure (VINCI) VA Salt Lake City Health Care System, 500 Foothill Drive, Salt Lake City, UT 84148
- Mary T. Brophy M.D., M.P.H., Director, VA Central Biorepository
  VA Boston Healthcare System, 150 S. Huntington Avenue, Boston, MA 02130
- MVP Coordinating Centers
  ○ MVP Coordinating Center, Boston—J. Michael Gaziano, M.D., M.P.H.
  ○ VA Boston Healthcare System, 150 S. Huntington Avenue, Boston, MA 02130
  ○ MVP Coordinating Center, Palo Alto—Philip S. Tsao, Ph.D.
  ○ VA Palo Alto Health Care System, 3801 Miranda Avenue, Palo Alto, CA 94304
  ○ MVP Information Center, Canandaigua—Brady Stephens, M.S.
  ○ Canandaigua VA Medical Center, 400 Fort Hill Avenue, Canandaigua, NY 14424
  ○ Cooperative Studies Program Clinical Research Pharmacy Coordinating Center, Albuquerque—Todd Connor, Pharm.D.; Dean P. Argyres, B.S., M.S.

New Mexico VA Health Care System, 1501 San Pedro Drive SE, Albuquerque, NM 87108

**MVP Publications and Presentations Committee**

- Co-Chair: Themistocles L. Assimes, M.D., Ph.D
  VA Palo Alto Health Care System, 3801 Miranda Avenue, Palo Alto, CA 94304
- Co-Chair: Adriana Hung, M.D.; M.P.H
  VA Tennessee Valley Healthcare System, 1310 24th Ave. South, Nashville, TN 37212
- Co-Chair: Henry Kranzler, M.D.
  Philadelphia VA Medical Center, 3900 Woodland Avenue, Philadelphia, PA 19104

**MVP Local Site Investigators**

- Samuel Aguayo, M.D., Phoenix VA Health Care System 650 E. Indian School Road, Phoenix, AZ 85012
- Sunil Ahuja, M.D., South Texas Veterans Health Care System 7400 Merton Minter Boulevard, San Antonio, TX 78229
- Kathrina Alexander, M.D., Veterans Health Care System of the Ozarks 1100 North College Avenue, Fayetteville, AR 72703
- Xiao M. Androulakis, M.D., Columbia VA Health Care System 6439 Garners Ferry Road, Columbia, SC 29209
- Prakash Balasubramanian, M.D., William S. Middleton Memorial Veterans Hospital 2500 Overlook Terrace, Madison, WI 53705
- Zuhair Ballas, M.D., Iowa City VA Health Care System 601 Highway 6 West, Iowa City, IA 52246-2208
- Jean Beckham, Ph.D., Durham VA Medical Center 508 Fulton Street, Durham, NC 27705
- Sujata Bhushan, M.D., VA North Texas Health Care System 4500 S. Lancaster Road, Dallas, TX 75216
- Edward Boyko, M.D., VA Puget Sound Health Care System 1660 S. Columbian Way, Seattle, WA 98108-1597
- David Cohen, M.D., Portland VA Medical Center
  3710 SW U.S. Veterans Hospital Road, Portland, OR 97239
- Louis Dellitalia, M.D., Birmingham VA Medical Center 700 S. 19th Street, Birmingham AL 35233
- L. Christine Faulk, M.D., Robert J. Dole VA Medical Center 5500 East Kellogg Drive, Wichita, KS 67218-1607
- Joseph Fayad, M.D., VA Southern Nevada Healthcare System 6900 North Pecos Road, North Las Vegas, NV 89086
- Daryl Fujii, Ph.D., VA Pacific Islands Health Care System 459 Patterson Rd, Honolulu, HI 96819
- Saib Gappy, M.D., John D. Dingell VA Medical Center 4646 John R Street, Detroit, MI 48201
- Frank Gesek, Ph.D., White River Junction VA Medical Center 163 Veterans Drive, White River Junction, VT 05009
- Jennifer Greco, M.D., Sioux Falls VA Health Care System 2501 W 22nd Street, Sioux Falls, SD 57105
- Michael Godschalk, M.D., Richmond VA Medical Center 1201 Broad Rock Blvd., Richmond, VA 23249
- Todd W. Gress, M.D., Ph.D., Hershel “Woody” Williams VA Medical Center 1540 Spring Valley Drive, Huntington, WV 25704
- Samir Gupta, M.D., M.S.C.S., VA San Diego Healthcare System 3350 La Jolla Village Drive, San Diego, CA 92161
- Salvador Gutierrez, M.D., Edward Hines, Jr. VA Medical Center 5000 South 5th Avenue, Hines, IL 60141
- John Harley, M.D., Ph.D., Cincinnati VA Medical Center 3200 Vine Street, Cincinnati, OH 45220
- Kimberly Hammer, Ph.D., Fargo VA Health Care System 2101 N. Elm, Fargo, ND 58102
- Mark Hamner, M.D., Ralph H. Johnson VA Medical Center 109 Bee Street, Mental Health Research, Charleston, SC 29401
- Adriana Hung, M.D., M.P.H., VA Tennessee Valley Healthcare System 1310 24th Avenue, South Nashville, TN 37212
- Robin Hurley, M.D., W.G. (Bill) Hefner VA Medical Center 1601 Brenner Ave, Salisbury, NC 28144
- Pran Iruvanti, D.O., Ph.D., Hampton VA Medical Center 100 Emancipation Drive, Hampton, VA 23667
- Frank Jacono, M.D., VA Northeast Ohio Healthcare System 10701 East Boulevard, Cleveland, OH 44106
- Darshana Jhala, M.D., Philadelphia VA Medical Center 3900 Woodland Avenue, Philadelphia, PA 19104
- Scott Kinlay, M.B.B.S., Ph.D., VA Boston Healthcare System 150 S. Huntington Avenue, Boston, MA 02130
- Jon Klein, M.D., Ph.D., Louisville VA Medical Center 800 Zorn Avenue, Louisville, KY 40206
- Michael Landry, Ph.D., Southeast Louisiana Veterans Health Care System 2400 Canal Street, New Orleans, LA 70119
- Peter Liang, M.D., M.P.H., VA New York Harbor Healthcare System 423 East 23rd Street, New York, NY 10010
- Suthat Liangpunsakul, M.D., M.P.H., Richard Roudebush VA Medical Center 1481 West 10th Street, Indianapolis, IN 46202
- Jack Lichy, M.D., Ph.D., Washington DC VA Medical Center 50 Irving St, Washington, D. C. 20422
- C. Scott Mahan, M.D., Charles George VA Medical Center 1100 Tunnel Road, Asheville, NC 28805
- Ronnie Marrache, M.D., VA Maine Healthcare System 1 VA Center, Augusta, ME 04330
- Stephen Mastorides, M.D., James A. Haley Veterans’ Hospital 13000 Bruce B. Downs Blvd, Tampa, FL 33612
- Elisabeth Mates M.D., Ph.D., VA Sierra Nevada Health Care System 975 Kirman Avenue, Reno, NV 89502
- Kristin Mattocks, Ph.D., M.P.H., Central Western Massachusetts Healthcare System 421 North Main Street, Leeds, MA 01053
- Paul Meyer, M.D., Ph.D., Southern Arizona VA Health Care System 3601 S 6th Avenue, Tucson, AZ 85723
- Jonathan Moorman, M.D., Ph.D., James H. Quillen VA Medical Center Corner of Lamont & Veterans Way, Mountain Home, TN 37684
- Timothy Morgan, M.D., VA Long Beach Healthcare System 5901 East 7th Street Long Beach, CA 90822
- Maureen Murdoch, M.D., M.P.H., Minneapolis VA Health Care System One Veterans Drive, Minneapolis, MN 55417
- James Norton, Ph.D., VA Health Care Upstate New York 113 Holland Avenue, Albany, NY 12208
- Olaoluwa Okusaga, M.D., Michael E. DeBakey VA Medical Center 2002 Holcombe Blvd, Houston, TX 77030
- Kris Ann Oursler, M.D., Salem VA Medical Center 1970 Roanoke Blvd, Salem, VA 24153
- Ana Palacio, M.D., M.P.H., Miami VA Health Care System 1201 NW 16th Street, 11 GRC, Miami FL 33125
- Samuel Poon, M.D., Manchester VA Medical Center 718 Smyth Road, Manchester, NH 03104
- Emily Potter, Pharm.D., VA Eastern Kansas Health Care System 4101 S 4th Street Trafficway, Leavenworth, KS 66048
- Michael Rauchman, M.D., St. Louis VA Health Care System 915 North Grand Blvd, St. Louis, MO 63106
- Richard Servatius, Ph.D., Syracuse VA Medical Center 800 Irving Avenue, Syracuse, NY 13210
- Satish Sharma, M.D., Providence VA Medical Center 830 Chalkstone Avenue, Providence, RI 02908
- River Smith, Ph.D., Eastern Oklahoma VA Health Care System 1011 Honor Heights Drive, Muskogee, OK 74401
- Peruvemba Sriram, M.D., N. FL/S. GA Veterans Health System 1601 SW Archer Road, Gainesville, FL 32608
- Patrick Strollo, Jr., M.D., VA Pittsburgh Health Care System University Drive, Pittsburgh, PA 15240
- Neeraj Tandon, M.D., Overton Brooks VA Medical Center 510 East Stoner Ave, Shreveport, LA 71101
- Philip Tsao, Ph.D., VA Palo Alto Health Care System 3801 Miranda Avenue, Palo Alto, CA 94304-1290
- Gerardo Villareal, M.D., New Mexico VA Health Care System 1501 San Pedro Drive, S.E. Albuquerque, NM 87108
- Agnes Wallbom, M.D., M.S., VA Greater Los Angeles Health Care System 11301 Wilshire Blvd, Los Angeles, CA 90073
- Jessica Walsh, M.D., VA Salt Lake City Health Care System 500 Foothill Drive, Salt Lake City, UT 84148
- John Wells, Ph.D., Edith Nourse Rogers Memorial Veterans Hospital 200 Springs Road, Bedford, MA 01730
- Jeffrey Whittle, M.D., M.P.H., Clement J. Zablocki VA Medical Center 5000 West National Avenue, Milwaukee, WI 53295
- Mary Whooley, M.D., San Francisco VA Health Care System 4150 Clement Street, San Francisco, CA 94121
- Allison E. Williams, N.D., Ph.D., R.N, Bay Pines VA Healthcare System 10,000 Bay Pines Blvd Bay Pines, FL 33744
- Peter Wilson, M.D., Atlanta VA Medical Center 1670 Clairmont Road, Decatur, GA 30033
- Junzhe Xu, M.D., VA Western New York Healthcare System 3495 Bailey Avenue, Buffalo, NY 14215-1199

Shing Shing Yeh, Ph.D., M.D., Northport VA Medical Center 79 Middleville Road, Northport, NY 11768

## References

[1] Fukuda K., Nisenbaum R., Stewart G., Thompson W.W., Robin L., Washko R.M., Noah D.L., Barrett D.H., Randall B., Herwaldt B.L. (1998). Chronic multisymptom illness affecting Air Force veterans of the Gulf War. JAMA.

[2] Blanchard M.S., Eisen S.A., Alpern R., Karlinsky J., Toomey R., Reda D.J., Murphy F.M., Jackson L.W., Kang H.K. (2006). Chronic multisymptom illness complex in Gulf War I veterans 10 years later. Am. J. Epidemiol..

[3] Gwini S.M., Forbes A.B., Sim M.R., Kelsall H.L. (2016). Multisymptom Illness in Gulf War Veterans: A Systematic Review and Meta-Analysis. J. Occup. Environ. Med..

[4] Porter B., Long K., Rull R.P., Dursa E.K., Millennium Cohort Study Team (2020). Prevalence of Chronic Multisymptom Illness/Gulf War Illness Over Time Among Millennium Cohort Participants, 2001 to 2016. J. Occup. Environ. Med..

[5] Steele L. (2000). Prevalence and patterns of Gulf War illness in Kansas veterans: Association of symptoms with characteristics of person, place, and time of military service. Am. J. Epidemiol..

[6] Kang H.K., Mahan C.M., Lee K.Y., Magee C.A., Murphy F.M. (2000). Illnesses among United States veterans of the Gulf War: A population-based survey of 30,000 veterans. J. Occup. Environ. Med..

[7] Research Advisory Committee on Gulf War Veterans’ Illness (2008). Gulf War Illness and the Health of Gulf War Veterans: Scientific Findings and Recommendations.

[8] Institute of Medicine (IOM) Committee on Gulf War and Health (2010). Update of Health Effects of Serving in the Gulf War. Gulf War and Health.

[9] Dursa E.K., Cao G., Porter B., Culpepper W.J., Schneiderman A.I. (2021). The Health of Gulf War and Gulf Era Veterans Over Time: U.S. Department of Veterans Affairs’ Gulf War Longitudinal Study. J. Occup. Environ. Med..

[10] Gifford E.J., Boyle S.H., Vahey J., Sims K.J., Efird J.T., Chesnut B., Stafford C., Upchurch J., Williams C.D., Helmer D.A. (2022). Health-Related Quality of Life by Gulf War Illness Case Status. Int. J. Environ. Res. Public Health.

[11] Khalil L., McNeil R.B., Sims K.J., Felder K.A., Hauser E.R., Goldstein K.M., Voils C.I., Klimas N.G., Brophy M.T., Thomas C.M. (2018). The Gulf War Era Cohort and Biorepository: A Longitudinal Research Resource of Veterans of the 1990–1991 Gulf War Era. Am. J. Epidemiol..

[12] Research Advisory Committee on Gulf War Veterans’ Illnesses (2014). Gulf War Illness and the Health of Gulf War Veterans: Research Update and Recommendations, 2009–2013.

[13] Haley R.W., Kramer G., Xiao J., Dever J.A., Teiber J.F. (2022). Evaluation of a Gene-Environment Interaction of PON1 and Low-Level Nerve Agent Exposure with Gulf War Illness: A Prevalence Case-Control Study Drawn from the U.S. Military Health Survey’s National Population Sample. Environ. Health Perspect..

[14] Steele L., Lockridge O., Gerkovich M.M., Cook M.R., Sastre A. (2015). Butyrylcholinesterase genotype and enzyme activity in relation to Gulf War illness: Preliminary evidence of gene-exposure interaction from a case-control study of 1991 Gulf War veterans. Environ. Health.

[15] Vahey J., Gifford E.J., Sims K.J., Chesnut B., Boyle S.H., Stafford C., Upchurch J., Stone A., Pyarajan S., Efird J.T. (2021). Gene-Toxicant Interactions in Gulf War Illness: Differential Effects of the PON1 Genotype. Brain Sci..

[16] Nugent S.M., Freeman M., Ayers C.K., Winchell K.A., Press A.M., O’Neil M.E., Paynter R., Kansagara D. (2021). A Systematic Review of Therapeutic Interventions and Management Strategies for Gulf War Illness. Mil. Med..

[17] Gean E.G., Ayers C.K., Winchell K.A., Freeman M., Press A.M., Paynter R., Kansagara D., Nugent S.M. (2021). Biological measures and diagnostic tools for Gulf War Illness—A systematic review. Life Sci..

[18] Gaziano J.M., Concato J., Brophy M., Fiore L., Pyarajan S., Breeling J., Whitbourne S., Deen J., Shannon C., Humphries D. (2016). Million Veteran Program: A mega-biobank to study genetic influences on health and disease. J. Clin. Epidemiol..

[19] Nguyen X.-M.T., Whitbourne S.B., Li Y., Quaden R.M., Song R.J., Nguyen H.-N.A., Harrington K., Djousse L., Brewer J.V.V., Deen J. (2023). Data Resource Profile: Self-reported data in the Million Veteran Program: Survey development and insights from the first 850 736 participants. Int. J. Epidemiol..

[20] Radhakrishnan K., Hauser E.R., Polimanti R., Helmer D.A., Provenzale D., McNeil R.B., Maffucci A., Quaden R., Zhao H., Whitbourne S.B. (2021). Genomics of Gulf War Illness in U.S. Veterans Who Served during the 1990–1991 Persian Gulf War: Methods and Rationale for Veterans Affairs Cooperative Study #2006. Brain Sci..

[21] Duong L.M., Nono Djotsa A.B.S., Vahey J., Steele L., Quaden R., Harrington K.M., Ahmed S.T., Polimanti R., Streja E., Gaziano J.M. (2022). Association of Gulf War Illness with Characteristics in Deployed vs. Non-Deployed Gulf War Era Veterans in the Cooperative Studies Program 2006/Million Veteran Program 029 Cohort: A Cross-Sectional Analysis. Int. J. Environ. Res. Public Health.

[22] Brown S.H., Lincoln M.J., Groen P.J., Kolodner R.M. (2003). VistA–U.S. Department of Veterans Affairs national-scale HIS. Int. J. Med. Inf..

[23] U.S. Department of Veterans Affairs (2023). Observational Medical Outcomes Partnership (OMOP). U.S. Department of Veterans Affairs. https://www.herc.research.va.gov/include/page.asp?id=omop.

[24] U.S. Department of Veterans Affairs, U.S. Department of Defense, United States Veterans Health Administration, United States Office of the Assistant Secretary of Defense (Health Affairs) (2002). Combined Analysis of the VA and DoD Gulf War Clinical Evaluation Programs: A Study of the Clinical Findings from Systematic Medical Examinations of 100,339 U.S. Gulf War Veterans.

[25] Hays R.D., Sherbourne C.D., Mazel R.M. (1993). The rand 36-item health survey 1.0. Health Econ..

[26] Kazis L.E., Miller D.R., Clark J.A., Skinner K.M., Lee A., Ren X.S., Spiro A., Rogers W.H., Ware J.E. (2004). Improving the Response Choices on the Veterans SF-36 Health Survey Role Functioning Scales: Results from the Veterans Health Study. J. Ambul. Care Manag..

[27] Iqbal S.U., Rogers W., Selim A., Qian S., Lee A., Ren X.S., Rothendler J., Miller D., Kazis L.E., Center for Health Quality, Outcomes and Economic Research (2007). The Veterans RAND 12 Item Health Survey (VR-12): What It Is and How it is Used.

[28] Bush K., Kivlahan D.R., McDonell M.B., Fihn S.D., Bradley K.A. (1998). The AUDIT alcohol consumption questions (AUDIT-C): An effective brief screening test for problem drinking. Ambulatory Care Quality Improvement Project (ACQUIP). Alcohol. Use Disord. Identif. Test. Arch. Intern. Med..

[29] Quan H., Li B., Couris C.M., Fushimi K., Graham P., Hider P., Januel J.M., Sundararajan V. (2011). Updating and validating the Charlson comorbidity index and score for risk adjustment in hospital discharge abstracts using data from 6 countries. Am. J. Epidemiol..

[30] Nguyen X.T., Quaden R.M., Song R.J., Ho Y.L., Honerlaw J., Whitbourne S., DuVall S.L., Deen J., Pyarajan S., Moser J. (2018). Baseline Characterization and Annual Trends of Body Mass Index for a Mega-Biobank Cohort of US Veterans 2011–2017. J. Health Res. Rev. Dev. Ctries..

[31] Austin P.C. (2009). Balance diagnostics for comparing the distribution of baseline covariates between treatment groups in propensity-score matched samples. Stat. Med..

[32] Austin P.C. (2009). Using the Standardized Difference to Compare the Prevalence of a Binary Variable Between Two Groups in Observational Research. Commun. Stat. Simul. Comput..

[33] Yang D., Dalton J.E. (2012). A Unified Approach to Measuring the Effect Size between Two Group Using SAS. SAS Glob. Forum.

[34] Sullivan G.M., Feinn R. (2012). Using Effect Size—Or Why the P Value Is Not Enough. J. Grad. Med. Educ..

[35] Andrade C. (2020). Mean Difference, Standardized Mean Difference (SMD), and Their Use in Meta-Analysis: As Simple as It Gets. J. Clin. Psychiatry.

[36] Whitbourne S.B., Nguyen X.-M.T., Song R.J., Lord E., Lyden M., Harrington K.M., Ward R., Li Y., Brewer J.V.V., Cho K. (2022). Million Veteran Program’s response to COVID-19: Survey development and preliminary findings. PLoS ONE.

[37] Davis K.A.S., Coleman J.R.I., Adams M., Allen N., Breen G., Cullen B., Dickens C., Fox E., Graham N., Holliday J. (2020). Mental health in UK Biobank—Development, implementation and results from an online questionnaire completed by 157 366 participants: A reanalysis. BJPsych Open.

[38] Kang H.K., Li B., Mahan C.M., Eisen S.A., Engel C.C. (2009). Health of US Veterans of 1991 Gulf War: A Follow-Up Survey in 10 Years. J. Occup. Environ. Med..

[39] Fry A., Littlejohns T.J., Sudlow C., Doherty N., Adamska L., Sprosen T., Collins R., Allen N.E. (2017). Comparison of Sociodemographic and Health-Related Characteristics of UK Biobank Participants with Those of the General Population. Am. J. Epidemiol..

[40] Hillyer G.C., Park Y.A., Rosenberg T.H., Mundi P., Patel I., Bates S.E. (2021). Positive attitudes toward clinical trials among military veterans leaves unanswered questions about poor trial accrual. Semin. Oncol..

[41] Campbell H.M., Raisch D.W., Sather M.R., Warren S.R., Segal A.R. (2007). A comparison of veteran and nonveteran motivations and reasons for participating in clinical trials. Mil. Med..

[42] Grewe M.E., Khalil L., Felder K., Goldstein K.M., McNeil R.B., Sims K.J., Provenzale D., Voils C.I. (2021). Gulf War Era Veterans’ perspectives on research: A qualitative study. Life Sci..

[43] Andreeva V.A., Salanave B., Castetbon K., Deschamps V., Vernay M., Kesse-Guyot E., Hercberg S. (2015). Comparison of the sociodemographic characteristics of the large NutriNet-Santé e-cohort with French Census data: The issue of volunteer bias revisited. J. Epidemiol. Commun. Health.

[44] Mishra G.D., Hockey R., Powers J., Loxton D., Tooth L., Rowlands I., Byles J., Dobson A. (2014). Recruitment via the Internet and social networking sites: The 1989–1995 cohort of the Australian Longitudinal Study on Women’s Health. J. Med. Internet. Res..

[45] Jensen M.D., Ryan D.H., Apovian C.M., Ard J.D., Comuzzie A.G., Donato K.A., Hu F.B., Hubbard V.S., Jakicic J.M., Kushner R.F. (2014). 2013 AHA/ACC/TOS guideline for the management of overweight and obesity in adults: A report of the American College of Cardiology/American Heart Association Task Force on Practice Guidelines and The Obesity Society. Circulation.

[46] Li M., Gong W., Wang S., Li Z. (2022). Trends in body mass index, overweight and obesity among adults in the USA, the NHANES from 2003 to 2018: A repeat cross-sectional survey. BMJ Open.

[47] Zundel C.G., Heeren T., Grasso C.M., Spiro A., Proctor S.P., Sullivan K., Krengel M. (2020). Changes in Health Status in the Ft. Devens Gulf War. Veterans Cohort: 1997–2017. Neurosci. Insights.

[48] Koller D.C.-M.B., Nono Djotsa A., Wendt F., De Lillo A., Friligkou E., Polimanti R. (2024). Multi-ancestry Genome-wide Association Study of Gulf War Illness Research Definitions in the Million Veteran Program (under review).

