# Supplementary material for: The Million Veteran Program 1990–1991 Gulf War Era Survey: An Evaluation of Veteran Response, Characteristics, and Representativeness of the Gulf War Era Veteran Population
Source: Int J Environ Res Public Health. 2024 Jan 8;21(1):72. doi: 10.3390/ijerph21010072 (PMC10815483; doi:10.3390/ijerph21010072)
Supplement: Supplementary file 1 [file ijerph-21-00072-s001.zip › Supplemental File S1_MVP 1990-1991 Gulf War Era Survey_v2.pdf]

1990-1991 GULF WAR ERA SURVEY

The following questions ask about your military experience during the 1990-1991 Gulf War Era, regardless of whether you were deployed or not; the questions also ask about your health history and medication use.

By completing all or some of these questions, you are voluntarily consenting to participate in this survey.

Questionnaire Instructions:

We would like you to answer all of the questions on the following pages as completely as possible.

You are free to skip questions without any penalty or prejudice.

- Please fill in the circle completely.

  Shade circles like this --> ●

  Not like this --> ⊗ ✓

- It is best to use black or blue ink pen when completing this questionnaire. Please do not use a pencil, marker, or felt tip pen.
- If you are unsure about how to answer a question, please give the best answer you can.
- Answer each question, unless you are asked to skip to another question.
- Fill in only one answer circle for each question, unless it tells you to "Fill in all that apply."
- Please do not draw a line through any boxes that are left blank or not applicable.
- When you are finished, please place the questionnaire in the enclosed postage-paid envelope and place in the mail.

1. **What is today's date?**

|__|__| / |__|__| / |__|__|__|__|

mm dd yyyy

2. **What is your date of birth?**

|__|__| / |__|__| / |__|__|__|__|

mm dd yyyy

3. **What is your gender?**

- ○ Male
- ○ Female

4. **Are you Spanish, Hispanic, or Latino?** *(Fill in all that apply)*

- ○ No, not Spanish, Hispanic, Latino
- ○ Yes, Mexican, Mexican American, Chicano
- ○ Yes, Puerto Rican
- ○ Yes, Cuban
- ○ Yes, other Spanish, Hispanic, Latino

5. **What is your race?** *(Fill in all that apply)*

- ○ White
- ○ Black/African-American
- ○ American Indian/Alaska Native
- ○ Chinese
- ○ Japanese
- ○ Asian Indian
- ○ Other Asian
- ○ Filipino
- ○ Pacific Islander
- ○ Other: ____________________

6. **Where are your ancestors originally from?** *(Fill in all that apply)*

- ○ Africa
- ○ East Asia/Pacific Ocean region
- ○ Middle East
- ○ North America
- ○ Northern Europe
- ○ Southern Europe
- ○ South America
- ○ Southwest Asia
- ○ (Don't Know)

7. **Did you serve in the military during the 1990-1991 Gulf War Era?**

- ○ Yes
- ○ No → **Please stop and return the questionnaire in the enclosed envelope. Thank you!**

8. **During the 1990-1991 Gulf War Era, which branch(es) of the military did you serve?**
(Fill in all that apply)

- ○ Army
- ○ Navy
- ○ Air Force
- ○ Marine Corps
- ○ Coast Guard
- ○ National Guard
- ○ Merchant Marines
- ○ NOAA
- ○ Public Health Services

9. **Did you deploy at any time to the Persian Gulf Region during the 1990-1991 Gulf War Era?**

- ○ Yes
- ○ No → **Skip to question 14**

10. **In what month and year did you first arrive in the Persian Gulf Region?**

|__|__| / |__|__|__|__|

Month Year

11. **In what month and year did you last leave the Persian Gulf Region?**

|__|__| / |__|__|__|__|

Month Year

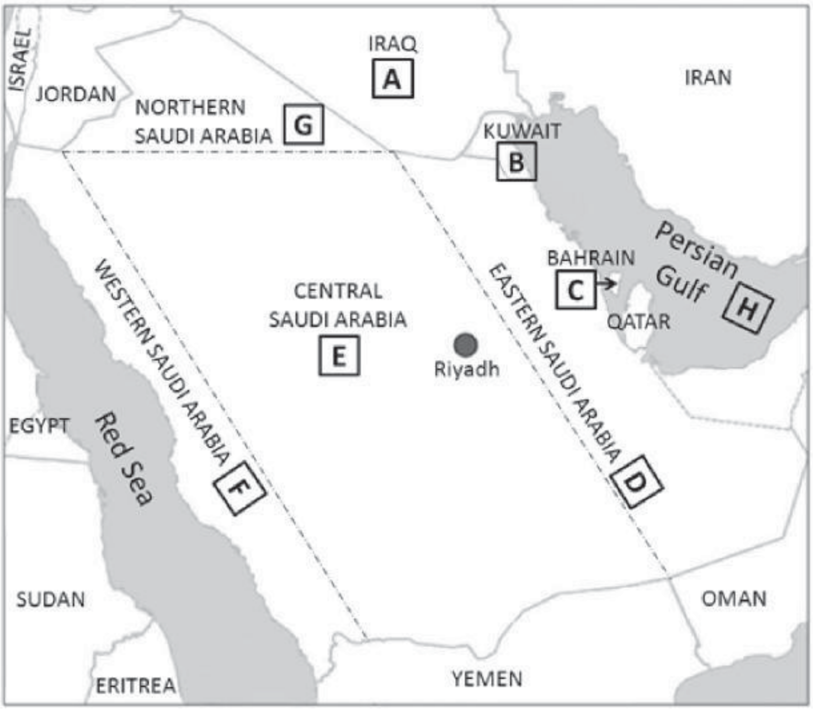

12. While you were in the Persian Gulf Region, were you ever located in...? (*Fill in No or Yes for each*)	No	Yes	IF YES: About how many days? 1-6 days	7-30 days	31 days or more
a. Iraq (area A on map)	○ No	○ Yes ➔	○	○	○
b. Kuwait (area B on map)	○ No	○ Yes ➔	○	○	○
c. Bahrain (area C on map)	○ No	○ Yes ➔	○	○	○
d. Saudi Arabia: Eastern area (area D on map)	○ No	○ Yes ➔	○	○	○
e. Saudi Arabia: Central area (area E on map)	○ No	○ Yes ➔	○	○	○
f. Saudi Arabia: Western area (area F on map)	○ No	○ Yes ➔	○	○	○
g. Saudi Arabia: Northern area (area G on map)	○ No	○ Yes ➔	○	○	○
h. At sea: in the Persian Gulf (area H on map)	○ No	○ Yes ➔	○	○	○
i. At sea: other location ______	○ No	○ Yes ➔	○	○	○
j. Other location ______	○ No	○ Yes ➔	○	○	○

13. While you were in the Persian Gulf Region, did you experience any of the following?	Not Sure	No	Yes	IF YES: About how many days? 1-6 days	7-30 days	31 days or more
a. Entered Iraq	○ Not Sure	○ No	○ Yes →	○	○	○
b. Entered Kuwait	○ Not Sure	○ No	○ Yes →	○	○	○
c. Served on board a ship	○ Not Sure	○ No	○ Yes →	○	○	○
d. Close proximity to smoke from oil well fires	○ Not Sure	○ No	○ Yes →	○	○	○
e. Directly involved in ground combat	○ Not Sure	○ No	○ Yes →	○	○	○
f. Took pyridostigmine bromide (anti-nerve agent pills)	○ Not Sure	○ No	○ Yes →	○	○	○
g. Exposed to chemical or biological warfare agents	○ Not Sure	○ No	○ Yes →	○	○	○
h. Worked with prisoners of war	○ Not Sure	○ No	○ Yes →	○	○	○
i. Used pesticide cream or liquid on your skin	○ Not Sure	○ No	○ Yes →	○	○	○
j. Wore a uniform treated with pesticides	○ Not Sure	○ No	○ Yes →	○	○	○
k. Used insect baits/no-pest strips in your living area	○ Not Sure	○ No	○ Yes →	○	○	○

14. Did you deploy in support of Operation Enduring Freedom (OEF) or Operation Iraqi Freedom (OIF) (September 2001 or later)?

- O Yes
- O No

15. Were you ever exposed to Agent Orange?

- O Yes
- O No
- O Not Sure

16. Were you ever given the Anthrax vaccine?

- O Yes
- O No
- O Not Sure

Health and Other Status

17. What is the highest degree or level of school you have completed?

- O Less than high school
- O High school diploma/GED
- O Some college credit, but no degree
- O Associate's degree (e.g., AA, AS)
- O Bachelor's degree (e.g., BA, BS)
- O Master's degree (e.g., MA, MS, MBA)
- O Professional or Doctorate degree

18. What is your current marital status?

- O Married
- O Civil commitment
- O Cohabitating
- O Separated
- O Divorced
- O Widowed
- O Never married

19. Including yourself, how many people currently live in your household?

1	2	3	4	5	6	7	8	9+
O	O	O	O	O	O	O	O	O

20a. In the PAST YEAR, about how much of your health care did you get at a VA facility (e.g., doctor's visits, hospitalizations, urgent care visits or counseling)?

- O None
- O 1 – 25%
- O 26 – 50%
- O 51 – 75%
- O 76 – 99%
- O 100%

20b. In the PAST YEAR, how many times were you a patient in a VA Healthcare Facility overnight or longer?

- O None
- O 1 – 3
- O 4 – 6
- O 7 – 9
- O 10 or more

20c. In the PAST YEAR, how many times were you a patient in a Non-VA Healthcare Facility overnight or longer?

- O None
- O 1 – 3
- O 4 – 6
- O 7 – 9
- O 10 or more

21. In general, would you say your health is:

- O Excellent
- O Very good
- O Good
- O Fair
- O Poor

The following question is about activities you might do during a typical day.

22. Does your health now limit you in these activities? If so, how much?

a. Moderate activities, such as moving a table, pushing a vacuum cleaner, bowling, or playing golf:

- O Yes, limited a lot
- O Yes, limited a little
- O No, not limited at all

b. Climbing several flights of stairs:

- O Yes, limited a lot
- O Yes, limited a little
- O No, not limited at all

23. During the PAST 4 WEEKS, have you had any of the following problems with your work or other regular daily activities as a result of your physical health?

a. Accomplished less than you would like:

- O No, none of the time
- O Yes, a little of the time
- O Yes, some of the time
- O Yes, most of the time
- O Yes, all of the time

b. Were limited in the kind of work or other activities:

- O No, none of the time
- O Yes, a little of the time
- O Yes, some of the time
- O Yes, most of the time
- O Yes, all of the time

24. During the PAST 4 WEEKS, have you had any of the following problems with your work or other regular daily activities as a result of any emotional problems (such as feeling depressed or anxious)?

a. Accomplished less than you would like:

- O No, none of the time
- O Yes, a little of the time
- O Yes, some of the time
- O Yes, most of the time
- O Yes, all of the time

b. Did not do work or other activities as carefully as usual:

- O No, none of the time
- O Yes, a little of the time
- O Yes, some of the time
- O Yes, most of the time
- O Yes, all of the time

25. During the PAST 4 WEEKS, how much did pain interfere with your normal work (including both work outside the home and housework)?

- O Not at all
- O A little bit
- O Moderately
- O Quite a bit
- O Extremely

Questions 26-27 are about how you feel and how things have been with you. For each question, please give the one answer that comes closest to the way you have been feeling.

26. How much of the time during the PAST 4 WEEKS:

a. Have you felt calm and peaceful?

- O All of the time
- O Most of the time
- O A good bit of the time
- O Some of the time
- O A little of the time
- O None of the time

b. Did you have a lot of energy?

- O All of the time
- O Most of the time
- O A good bit of the time
- O Some of the time
- O A little of the time
- O None of the time

c. Have you felt downhearted and blue?

- O All of the time
- O Most of the time
- O A good bit of the time
- O Some of the time
- O A little of the time
- O None of the time

27. During the PAST 4 WEEKS, how much of the time has your physical health or emotional problems interfered with your social activities (like visiting with friends, relatives, etc.)

- O All of the time
- O Most of the time
- O Some of the time
- O A little of the time
- O None of the time

Now we'd like to ask you some questions about how your health may have changed.

28. COMPARED TO ONE YEAR AGO, how would you rate your:

a. Physical health in general now?

- O Much better
- O Slightly better
- O About the same
- O Slightly worse
- O Much worse

b. Emotional problems (such as feeling anxious, depressed or irritable) now?

- O Much better
- O Slightly better
- O About the same
- O Slightly worse
- O Much worse

c. Cognitive (memory and thinking) health in general now?

- O Much better
- O Slightly better
- O About the same
- O Slightly worse
- O Much worse

29. On a scale of 0 - 10, where 0 means no pain and 10 means pain as bad as you can imagine, please rate your overall amount of pain in the PAST WEEK:

0	1	2	3	4	5	6	7	8	9	10
O	O	O	O	O	O	O	O	O	O	O
No Pain										Pain as bad as you can imagine

Activities and Habits

30. How would you rate your current physical fitness status?

- O Very good
- O Fairly good
- O Satisfactory
- O Fairly poor
- O Very poor

31. How physically strenuous is your work/job (paid and unpaid)?

- O Very light (mainly sitting)
- O Light (mainly walking)
- O Medium (lifting, carrying light loads)
- O Heavy manual work (climbing, carrying heavy loads)
- O Not applicable

Personal Habits

32. How often do you exercise vigorously enough to work up a sweat?

- O Daily
- O 5-6 times a week
- O 2-4 times a week
- O Once a week
- O 1-3 times a month
- O Rarely/Never

33. How often do you have a drink containing alcohol?

- O Never → **Skip to Question 36**
- O 1-3 days per month
- O 1 day per week
- O 2-3 days per week
- O 4-5 days per week
- O 6+ days per week

34. How many drinks containing alcohol do you have on a typical day when you are drinking?

- O 1 or 2
- O 3 or 4
- O 5 or 6
- O 7 to 9
- O 10 or more

35. How often do you have six or more drinks on one occasion?

- O Never
- O Less than monthly
- O Monthly
- O 2-3 times per week
- O 4+ times per week

36. In your lifetime, have you smoked a total of at least 100 cigarettes, cigars, or pipes?

- O No → **Skip to Question 37**
- O Yes →

a. Have you ever smoked daily or almost every day for at least 1 consecutive year?

- O Yes
- O No

b. Do you smoke now?

- O Yes, daily
- O Yes, occasionally
- O Not at all

**37. For each of the following, <u>other than what was prescribed to you</u>, have you ever used...?**	**No**	**Yes**	**IF YES: When did you use the substance? (Fill in all that apply)** Last 12 months	Prior to last 12 months
a. Sedatives	O No	O Yes →	O	O
b. Tranquilizers	O No	O Yes →	O	O
c. Painkillers (other than over-the-counter medications)	O No	O Yes →	O	O
d. Stimulants	O No	O Yes →	O	O
e. Marijuana	O No	O Yes →	O	O
f. Cocaine or Crack	O No	O Yes →	O	O
g. Hallucinogens	O No	O Yes →	O	O
h. Inhalants/Solvents	O No	O Yes →	O	O
i. Heroin	O No	O Yes →	O	O
j. Other drug ______	O No	O Yes →	O	O
k. Have you been hospitalized for alcohol or drug dependence in the last 5 years? If yes, when?	O No	O Yes →	Most Recent Year Hospitalized ☐☐☐☐	

38. **Indicate No or Yes for each. Have you had a persistent or recurring problem with...?**	**No**	**Yes**	**IF YES: How would you rate this problem?** Mild	Moderate	Severe	**Year of Onset**
**a.** Fatigue	○ No	○ Yes →	○	○	○	☐☐☐☐
**b.** Feeling unwell after physical exercise or exertion	○ No	○ Yes →	○	○	○	☐☐☐☐
**c.** Problems getting to sleep or staying asleep	○ No	○ Yes →	○	○	○	☐☐☐☐
**d.** Not feeling rested after you sleep	○ No	○ Yes →	○	○	○	☐☐☐☐
**e.** Pain in your joints	○ No	○ Yes →	○	○	○	☐☐☐☐
**f.** Stiffness in your joints	○ No	○ Yes →	○	○	○	☐☐☐☐
**g.** Pain in your muscles	○ No	○ Yes →	○	○	○	☐☐☐☐
**h.** Body pain, where you hurt all over	○ No	○ Yes →	○	○	○	☐☐☐☐
**i.** Headaches	○ No	○ Yes →	○	○	○	☐☐☐☐
**j.** Feeling dizzy, lightheaded, or faint	○ No	○ Yes →	○	○	○	☐☐☐☐
**k.** Eyes very sensitive to light	○ No	○ Yes →	○	○	○	☐☐☐☐
**l.** Blurred or double vision	○ No	○ Yes →	○	○	○	☐☐☐☐
**m.** Numbness or tingling in your extremities	○ No	○ Yes →	○	○	○	☐☐☐☐
**n.** Tremors or shaking	○ No	○ Yes →	○	○	○	☐☐☐☐
**o.** Low tolerance for heat or cold	○ No	○ Yes →	○	○	○	☐☐☐☐
**p.** Night sweats	○ No	○ Yes →	○	○	○	☐☐☐☐
**q.** Having physical or mental symptoms in response to certain smells or chemicals	○ No	○ Yes →	○	○	○	☐☐☐☐
**r.** Skin rashes	○ No	○ Yes →	○	○	○	☐☐☐☐
**s.** Other skin problems ☐	○ No	○ Yes →	○	○	○	☐☐☐☐

38. **Indicate No or Yes for each. Have you had a persistent or recurring problem with...?**	**No**	**Yes**	**IF YES: How would you rate this problem?** Mild	Moderate	Severe	**Year of Onset**
**t.** Diarrhea	○ No	○ Yes ➔	○	○	○	☐☐☐☐
**u.** Nausea or upset stomach	○ No	○ Yes ➔	○	○	○	☐☐☐☐
**v.** Abdominal pain or cramping	○ No	○ Yes ➔	○	○	○	☐☐☐☐
**w.** Difficulty breathing or shortness of breath	○ No	○ Yes ➔	○	○	○	☐☐☐☐
**x.** Frequent coughing when you don't have a cold	○ No	○ Yes ➔	○	○	○	☐☐☐☐
**y.** Wheezing in your chest	○ No	○ Yes ➔	○	○	○	☐☐☐☐
**z.** Sore throat	○ No	○ Yes ➔	○	○	○	☐☐☐☐
**aa.** Tender or swollen glands (lymph nodes) in your neck or armpits	○ No	○ Yes ➔	○	○	○	☐☐☐☐
**bb.** Difficulty concentrating	○ No	○ Yes ➔	○	○	○	☐☐☐☐
**cc.** Difficulty remembering recent information	○ No	○ Yes ➔	○	○	○	☐☐☐☐
**dd.** Trouble finding words when speaking	○ No	○ Yes ➔	○	○	○	☐☐☐☐
**ee.** Feeling down or depressed	○ No	○ Yes ➔	○	○	○	☐☐☐☐
**ff.** Feeling irritable or having angry outbursts	○ No	○ Yes ➔	○	○	○	☐☐☐☐
**gg.** Feeling moody	○ No	○ Yes ➔	○	○	○	☐☐☐☐
**hh.** Feeling anxious	○ No	○ Yes ➔	○	○	○	☐☐☐☐

39. Please tell us if a doctor or other healthcare provider has ever told you that you have any of the following conditions. Fill in No or Yes for each. If Yes, write the year you were told, and whether you currently take any medication(s) ("Currently Taking Meds") for that condition.

Have you ever been told that you have... ?

39a. Circulatory System Problems	No	Yes	IF YES: Year Told	IF YES: Currently Taking Meds
1. High blood pressure (Hypertension)	O No	O Yes →	☐☐☐☐	O No O Yes
2. Stroke	O No	O Yes →	☐☐☐☐	O No O Yes
3. Transient ischemic attack (TIA)	O No	O Yes →	☐☐☐☐	O No O Yes
4. Heart attack	O No	O Yes →	☐☐☐☐	O No O Yes
5. Coronary artery/Coronary heart disease (includes angina)	O No	O Yes →	☐☐☐☐	O No O Yes
6. Peripheral vascular disease	O No	O Yes →	☐☐☐☐	O No O Yes
7. High cholesterol	O No	O Yes →	☐☐☐☐	O No O Yes
8. Pulmonary embolism or deep vein thrombosis (DVT)	O No	O Yes →	☐☐☐☐	O No O Yes
9. Congestive heart failure	O No	O Yes →	☐☐☐☐	O No O Yes
10. Other circulatory system problem ________	O No	O Yes →	☐☐☐☐	O No O Yes

Have you ever been told that you have... ?

39b. Skeletal/Muscular Problems	No	Yes	IF YES: Year Told	Currently Taking Meds
1. Osteoarthritis	○ No	○ Yes →		○ No ○ Yes
2. Rheumatoid arthritis	○ No	○ Yes →		○ No ○ Yes
3. Other arthritis	○ No	○ Yes →		○ No ○ Yes
4. Gout	○ No	○ Yes →		○ No ○ Yes
5. Osteoporosis	○ No	○ Yes →		○ No ○ Yes
6. Other skeletal/muscular problem	○ No	○ Yes →		○ No ○ Yes
**39c. Mental Health Disorders**				
1. Anxiety reaction/Panic disorder	○ No	○ Yes →		○ No ○ Yes
2. Attention deficit hyperactivity disorder (ADHD)	○ No	○ Yes →		○ No ○ Yes
3. Bipolar disorder	○ No	○ Yes →		○ No ○ Yes
4. Posttraumatic stress disorder (PTSD)	○ No	○ Yes →		○ No ○ Yes
5. Depression	○ No	○ Yes →		○ No ○ Yes
6. Eating disorder	○ No	○ Yes →		○ No ○ Yes
7. Personality disorder	○ No	○ Yes →		○ No ○ Yes
8. Schizophrenia	○ No	○ Yes →		○ No ○ Yes
9. Social phobia	○ No	○ Yes →		○ No ○ Yes
10.. Other mental health disorder	○ No	○ Yes →		○ No ○ Yes
11. Have you been hospitalized for posttraumatic stress disorder (PTSD) in the last 5 years? If yes, when?	○ No	○ Yes →	**Most Recent Year Hospitalized**	
12. Have you been hospitalized for depression in the last 5 years? If yes, when?	○ No	○ Yes →	**Most Recent Year Hospitalized**	
13. Have you been hospitalized for any other mental health disorder? If yes, when?	○ No	○ Yes →	**Most Recent Year Hospitalized**	

Have you ever been told that you have... ?

39d. Vision/Hearing Problems	No	Yes	IF YES: Year Told	Currently Taking Meds
1. Cataracts	○ No	○ Yes ➔		○ No ○ Yes
2. Glaucoma	○ No	○ Yes ➔		○ No ○ Yes
3. Macular degeneration	○ No	○ Yes ➔		○ No ○ Yes
4. Blindness, all causes	○ No	○ Yes ➔		○ No ○ Yes
5. Tinnitus, or ringing in the ears	○ No	○ Yes ➔		○ No ○ Yes
6. Severe hearing loss or partial deafness in one or both ears	○ No	○ Yes ➔		○ No ○ Yes
**39e. Infectious Diseases**				
1. Tuberculosis	○ No	○ Yes ➔		○ No ○ Yes
2. Hepatitis C	○ No	○ Yes ➔		○ No ○ Yes
3. HIV/AIDS	○ No	○ Yes ➔		○ No ○ Yes
4. Other infectious disease If yes, specify type:	○ No	○ Yes ➔		○ No ○ Yes
**39f. Kidney Diseases**				
1. Kidney disease without dialysis	○ No	○ Yes ➔		○ No ○ Yes
2. Kidney disease with dialysis	○ No	○ Yes ➔		○ No ○ Yes
3. Acute kidney disease with no current dialysis	○ No	○ Yes ➔		○ No ○ Yes

Have you ever been told that you have... ?

39g. Digestive System Problems	No	Yes	IF YES: Year Told	Currently Taking Meds
1. Acid reflux/GERD	○ No	○ Yes →		○ No ○ Yes
2. Peptic ulcers	○ No	○ Yes →		○ No ○ Yes
3. Bowel obstruction	○ No	○ Yes →		○ No ○ Yes
4. Colon polyps	○ No	○ Yes →		○ No ○ Yes
5. Irritable bowel syndrome (IBS)	○ No	○ Yes →		○ No ○ Yes
6. Ulcerative colitis	○ No	○ Yes →		○ No ○ Yes
7. Crohn's disease	○ No	○ Yes →		○ No ○ Yes
8. Celiac disease/Sprue	○ No	○ Yes →		○ No ○ Yes
9. Other digestive system disorder	○ No	○ Yes →		○ No ○ Yes
**39h. Cancer**				
1. Brain cancer	○ No	○ Yes →		○ No ○ Yes
2. Breast cancer	○ No	○ Yes →		○ No ○ Yes
3. Colon cancer/Rectal cancer	○ No	○ Yes →		○ No ○ Yes
4. Lung cancer	○ No	○ Yes →		○ No ○ Yes
5. Prostate cancer	○ No	○ Yes →		○ No ○ Yes
6. Melanoma	○ No	○ Yes →		○ No ○ Yes
7. Skin cancer, other than melanoma	○ No	○ Yes →		○ No ○ Yes
8. Other cancer	○ No	○ Yes →		○ No ○ Yes

Have you ever been told that you have... ?

39i. Nervous System Problems	No	Yes	IF YES: Year Told	Currently Taking Meds
1. Migraine headaches	○ No	○ Yes →		○ No ○ Yes
2. Other headaches	○ No	○ Yes →		○ No ○ Yes
3. Memory loss or impairment	○ No	○ Yes →		○ No ○ Yes
4. Dementia (includes Alzheimer's, vascular, etc.)	○ No	○ Yes →		○ No ○ Yes
5. Concussion or loss ofconsciousness	○ No	○ Yes →		○ No ○ Yes
6. Traumatic brain injury (TBI)	○ No	○ Yes →		○ No ○ Yes
7. Spinal cord injury or impairment	○ No	○ Yes →		○ No ○ Yes
8. Epilepsy/Seizure	○ No	○ Yes →		○ No ○ Yes
9. Parkinson's disease	○ No	○ Yes →		○ No ○ Yes
10. Amyotrophic lateral sclerosis (ALS) (Lou Gehrig's disease)	○ No	○ Yes →		○ No ○ Yes
11. Multiple sclerosis (MS)	○ No	○ Yes →		○ No ○ Yes
12. Other nervous system problem	○ No	○ Yes →		○ No ○ Yes

Have you ever been told that you have... ?

39j. Other Conditions	No	Yes	IF YES: Year Told	Currently Taking Meds
1. Asthma	○ No	○ Yes ➔		○ No ○ Yes
2. Chronic lung disease (COPD, Emphysema or Bronchitis)	○ No	○ Yes ➔		○ No ○ Yes
3. Diabetes/"Sugar"	○ No	○ Yes ➔		○ No ○ Yes
4. Enlarged prostate (Benign prostatic hyperplasia)	○ No	○ Yes ➔		○ No ○ Yes
5. Liver condition (e.g., Cirrhosis)	○ No	○ Yes ➔		○ No ○ Yes
6. Sleep apnea	○ No	○ Yes ➔		○ No ○ Yes
7. Lupus	○ No	○ Yes ➔		○ No ○ Yes
8. Thyroid problems	○ No	○ Yes ➔		○ No ○ Yes
9. Chronic Fatigue Syndrome	○ No	○ Yes ➔		○ No ○ Yes
10. Fibromyalgia	○ No	○ Yes ➔		○ No ○ Yes
11. Other disease/disorder a.		○ Yes ➔		○ No ○ Yes
b.		○ Yes ➔		○ No ○ Yes
c.		○ Yes ➔		○ No ○ Yes

39k. Gulf War Illness	No	Yes	IF YES: How would you rate this problem? Mild	Moderate	Severe	Year of Onset
1. Have you ever been told that you have Gulf War Illness?	○ No	○ Yes ➔	○	○	○	☐☐☐☐
2. Do you feel that you have Gulf War Illness?	○ No	○ Yes ➔	○	○	○	☐☐☐☐

40. Over the LAST 2 WEEKS, how often have you been bothered by any of the following problems?	Not at all	Several days	More than half the days	Nearly every day
a. Little interest or pleasure in doing things	○	○	○	○
b. Feeling down, depressed, or hopeless	○	○	○	○
c. Trouble falling or staying asleep, or sleeping too much	○	○	○	○
d. Feeling tired or having little energy	○	○	○	○
e. Poor appetite or overeating	○	○	○	○
f. Feeling bad about yourself - or that you are a failure or have let yourself or your family down	○	○	○	○
g. Trouble concentrating on things, such as reading the newspaper or watching television	○	○	○	○
h. Moving or speaking so slowly that other people could have noticed? Or the opposite - being so fidgety or restless that you have been moving around a lot more than usual	○	○	○	○

i. If you filled in *Several days, More than half the days, or Nearly every day* for any problems in the table above, how difficult have these problems made it for you to do your work, take care of things at home, or get along with other people?

- ○ Not difficult at all
- ○ Somewhat difficult
- ○ Very difficult
- ○ Extremely difficult

**41.** **Below is a list of problems and complaints that Veterans sometimes have in response to stressful life experiences. Please read each one carefully and fill in one bubble per row to indicate how much you have been bothered by that problem IN THE LAST MONTH.**	**Not at all**	**A little bit**	**Moder-ately**	**Quite a bit**	**Extremely**
**a.** Repeated, disturbing *memories*, *thoughts*, or *images* of a stressful experience from the past?	○	○	○	○	○
**b.** Repeated, disturbing *dreams* of a stressful experience from the past?	○	○	○	○	○
**c.** Suddenly *acting* or *feeling* as if a stressful experience *were happening* again (as if you were reliving it)?	○	○	○	○	○
**d.** Feeling *very upset* when *something reminded* you of a stressful experience from the past?	○	○	○	○	○
**e.** Having *physical reactions* (e.g., heart pounding, trouble breathing, or sweating) when *something reminded* you of a stressful experience from the past?	○	○	○	○	○
**f.** Avoid *thinking about* or *talking about* a stressful experience from the past or avoid *having feelings* related to it?	○	○	○	○	○
**g.** Avoid *activities* or *situations* because they *remind you* of a stressful experience from the past?	○	○	○	○	○
**h.** Trouble *remembering important parts* of a stressful experience from the past?	○	○	○	○	○
**i.** Loss of *interest in things that you used to enjoy*?	○	○	○	○	○
**j.** Feeling *distant* or *cut off* from other people?	○	○	○	○	○
**k.** Feeling *emotionally numb* or being unable to have loving feelings for those close to you?	○	○	○	○	○
**l.** Feeling as if your *future* will somehow be *cut short*?	○	○	○	○	○
**m.** Trouble *falling* or *staying asleep*?	○	○	○	○	○
**n.** Feeling *irritable* or having *angry outbursts*?	○	○	○	○	○
**o.** Having *difficulty concentrating*?	○	○	○	○	○
**p.** Being *"super alert"* or watchful on guard?	○	○	○	○	○
**q.** Feeling *jumpy* or easily startled?	○	○	○	○	○

Thank you for your time and participation in this study.

Thank you for your service!

Please return the completed questionnaire in the enclosed envelope to:

Department of Veterans Affairs

1990-1991 Gulf War Era Survey

PO Box 6378

Chicago, IL 60680-9919
