# Supplementary material for: The Million Veteran Program 1990–1991 Gulf War Era Survey: An Evaluation of Veteran Response, Characteristics, and Representativeness of the Gulf War Era Veteran Population
Source: Int J Environ Res Public Health. 2024 Jan 8;21(1):72. doi: 10.3390/ijerph21010072 (PMC10815483; doi:10.3390/ijerph21010072)
Supplement: Supplementary file 1 [file ijerph-21-00072-s001.zip › Supplemental File S2_GW Era Veteran Response Rates Stratified by Deployment Status and Rank_rev.pdf]

Supplementary File S2. Gulf War Era Veteran Response Rates Stratified by Deployment Status and Rank.

Category	Deployed		Not Deployed	
	**Officer (*n* = 2,180)**	**Enlisted (*n* = 21,108)**	**Officer (*n* = 12,731)**	**Enlisted (*n* = 69,880)**
Overall Response (*n*, %)	1,466 (67.3)	8,798 (41.7)	7,829 (61.5)	25,206 (36.1)
Gender[1] (%)				
Male	68.5	41.9	62.0	35.9
Female	61.6	39.6	59.0	36.8
Age Group[1] (%)				
40-49	48.5	29.7	41.3	23.2
50-59	58.3	38.4	51.7	29.5
60-69	71.0	53.5	62.2	44.3
70+	79.6	65.8	68.5	52.0
Race[1] (%)				
White	71.4	46.4	64.6	40.6
Black/African American	39.5	32.5	39.8	25.3
Asian	92.3	40.0	54.2	37.6
American Indian/Alaskan Native	58.5	39.8	50.3	34.1
Other	73.1	50.2	54.7	41.1
Multiple Responses	65.6	53.1	61.9	45.3
Missing	25.0	19.3	27.3	16.7
Ethnicity[1] (%)				
Hispanic or Latino	60.9	43.2	54.8	34.5
Not Hispanic or Latino	67.7	41.7	61.9	36.3
Unknown	--	12.8	35.0	14.6
Education[2] (%)				
High school or less	100.0	56.9	58.3	47.2
Some college	82.1	57.7	62.0	50.2
Associates	76.5	60.3	58.0	53.8
Bachelors	74.5	63.0	65.9	55.6
Masters/Professional/Doctorate	77.2	63.0	72.2	59.1
Missing	72.7	47.5	55.2	45.7
Income (annual)[2] (%)				
<$30,000	61.3	52.0	54.6	43.1
$30,000-$59,999	77.4	59.4	63.0	52.7
$60,000-$99,999	75.8	63.0	70.3	57.1
$100,000+	77.7	62.1	72.8	58.7
Missing	77.5	60.6	72.4	53.2
Marital Status[3] (%)				
Married or cohabitating with partner	77.2	60.3	70.6	53.6
Missing	29.7	17.3	24.0	12.4
Service Branch[4] (%)				
Army	66.6	41.3	59.4	34.4
Navy	68.8	39.8	64.4	35.1

Air force	68.1	49.2	63.5	42.7
Marine Corps	66.2	40.7	60.7	31.1
Coast Guard	75.0	71.4	67.5	39.1
Other[4]	29.7	17.3	24.0	12.4
Component[4] (%)				
Active Duty	66.5	40.3	64.4	35.8
National Guard	72.6	50.3	58.7	39.7
Reserves	68.0	46.5	57.9	34.5
Current health status[2] (%)				
Good to excellent	78.5	61.9	72.3	55.9
Fair to poor	70.7	56.5	61.6	47.1
Missing	60.0	42.0	48.0	40.4
Current physical fitness status[2] (%)				
Very good to fairly good	80.1	63.7	74.2	56.3
Satisfactory	73.9	59.7	67.5	53.1
Fairly poor to very poor	71.5	55.8	61.2	47.6
Missing	26.1	16.4	21.2	11.6
Current exercise frequency[2] (%)				
≤1–3 times/month	74.4	56.6	66.3	50.5
Once/week	73.4	61.9	66.9	53.3
2–4 times/week	76.9	62.2	71.9	54.4
≥ 5 times/week	82.5	59.9	74.8	54.5
Missing	57.1	53.9	56.9	39.0
Pain intensity, past week[2] (%)				
No pain (0)	80.0	61.4	73.2	56.0
Mild pain (1–3)	79.0	61.1	73.8	57.1
Moderate pain (4–6)	78.1	60.1	66.5	51.9
Severe pain (7–10)	63.2	56.8	58.3	47.3
Missing	--	55.8	66.7	44.1
VA health care use, past year[2] (%)				
None	79.3	64.7	72.7	59.1
Less than half of care	77.0	62.3	73.4	55.4
More than half of care	74.7	58.8	67.9	52.1
All care	75.7	56.6	65.3	49.5
Missing	26.4	16.9	21.3	12.0
VA inpatient hospital stays, past year[2] (n)				
None	77.5	60.6	71.1	54.3
1–3	72.3	53.1	60.7	45.5
≥ 4	60.0	49.8	51.8	38.4
Missing	72.1	59.0	69.7	51.2
Smoker[2]				
Never	73.3	51.4	64.5	44.2
Former	77.4	63.4	70.5	55.9
Current	76.9	60.1	70.6	53.4
Missing	66.7	52.7	71.6	48.4
Drinker- AUDIT-C [2,5] (%)				

Yes	80.3	58.5	73.5	53.4
No	75.2	59.8	69.0	52.6
Missing	26.9	17.5	22.3	12.7

Note: All numbers reported in table represent percents. For example, 68.5% of male deployed officers returned a GW Survey. [1] Data source for gender, age (calculated as of 1/1/1991), race, ethnicity from MVP Core demographics file supplemented with VADIR. [2] Data source used for education, income, and health characteristics was the MVP Baseline Survey. [3]Data source used for marital status was CDW. [4] Data source used for deployment, enlisted/officer, service branch, rank and component was VADIR. [5] Drinker - based on the AUDIT-C: defined as a positive screen for a score of 4 or more for males and a score of 3 or more for females.
